# Hypersensitive Response-Like Reaction Is Associated with Hybrid Necrosis in Interspecific Crosses between Tetraploid Wheat and *Aegilops tauschii* Coss

**DOI:** 10.1371/journal.pone.0011326

**Published:** 2010-06-25

**Authors:** Nobuyuki Mizuno, Naoki Hosogi, Pyoyun Park, Shigeo Takumi

**Affiliations:** 1 Laboratory of Plant Genetics, Graduate School of Agricultural Science, Kobe University, Kobe, Japan; 2 Laboratory of Stress Cytology, Graduate School of Agricultural Science, Kobe University, Kobe, Japan; University of Melbourne, Australia

## Abstract

**Background:**

Hybrid speciation is classified into homoploid and polyploid based on ploidy level. Common wheat is an allohexaploid species that originated from a naturally occurring interploidy cross between tetraploid wheat and diploid wild wheat *Aegilops tauschii* Coss. *Aegilops tauschii* provides wide naturally occurring genetic variation. Sometimes its triploid hybrids with tetraploid wheat show the following four types of hybrid growth abnormalities: types II and III hybrid necrosis, hybrid chlorosis, and severe growth abortion. The growth abnormalities in the triploid hybrids could act as postzygotic hybridization barriers to prevent formation of hexaploid wheat.

**Methodology/Principal Findings:**

Here, we report on the geographical and phylogenetic distribution of *Ae*. *tauschii* accessions inducing the hybrid growth abnormalities and showed that they are widely distributed across growth habitats in *Ae*. *tauschii*. Molecular and cytological characterization of the type III necrosis phenotype was performed. The hybrid abnormality causing accessions were widely distributed across growth habitats in *Ae. tauschii*. Transcriptome analysis showed that a number of defense-related genes such as pathogenesis-related genes were highly up-regulated in the type III necrosis lines. Transmission electron microscope observation revealed that cell death occurred accompanied by generation of reactive oxygen species in leaves undergoing type III necrosis. The reduction of photosynthetic activity occurred prior to the appearance of necrotic symptoms on the leaves exhibiting hybrid necrosis.

**Conclusions/Significance:**

Taking these results together strongly suggests that an autoimmune response might be triggered by intergenomic incompatibility between the tetraploid wheat and *Ae. tauschii* genomes in type III necrosis, and that genetically programmed cell death could be regarded as a hypersensitive response-like cell death similar to that observed in *Arabidopsis* intraspecific and *Nicotiana* interspecific hybrids. Only *Ae*. *tauschii* accessions without such inhibiting factors could be candidates for the D-genome donor for the present hexaploid wheat.

## Introduction

Hybrid speciation is classified into two types, homoploid and polyploid, based on ploidy level [1]. Homoploid speciation refers to the origin of a new hybrid lineage without a change in chromosome number, whereas polyploid speciation involves the full duplication of a hybrid genome. The Bateson-Dobzhansky-Muller (BDM) model simply explains the process for generating genetic incompatibilities in hybrids between two diverging lineages [2]. This model proposes that reduction of fitness in hybrids generally occurs due to interaction between at least two epistatic loci derived from divergent parents. A lot of BDM-type hybrid incompatibilities, including hybrid sterility and hybrid lethality, have been studied, and the molecular nature of the causal genes was recently elucidated in some animal and plant species [2], [3]. For example, it was reported that a nucleotide binding leucine rich repeat (NB-LRR)-type disease resistance (*R*) gene is both necessary and sufficient for induction of hybrid necrosis in intraspecific crosses of *Arabidopsis thaliana* [3], indicating that the hybrid necrosis is caused by particular alleles of the *R* locus inducing autoimmunity-like responses when epistatically interacted with particular alleles of genes elsewhere in the genome [3], [4]. In an interspecific cross of lettuce, *RIN4*, which encodes a protein interacting with multiple *R* gene products, is one of causal genes to introduce hybrid necrosis [4]. These successful studies suggested that hybrid incompatibility might arise as a by-product of adaptive evolution [3], because the genes causing hybrid incompatibility are rapidly evolving. Rapidly evolving gene families such as NB-LRR and F-box proteins are involved in sensing external inputs including pathogens and can similarly trigger programmed cell death [5].

Polyploid speciation is one of the most important evolutionary processes in plants and animals. Especially during the evolution of flowering plants, polyploid speciation events occurred frequently, because 47% to 70% of flowering plants are estimated to be descendants of polyploid progenitors [1]. A recent study revealed that a maternally expressed WRKY transcription factor controls hybrid lethality during seed development in interploidy crosses of *Arabidopsis* ecotypes [6]. Imprinted genes are also considered likely to be responsible for the hybrid lethality observed in interploidy crosses [7], [8]. However, it is largely unknown which genetic factors are involved in the postzygotic reproductive barriers for polyploid speciation.

Common wheat (*Triticum aestivum* L.) is an allohexaploid species (AABBDD genome) that originated from a naturally occurring interploidy cross between tetraploid wheat (*Triticum turgidum* L., AABB genome), including emmer and durum wheats, and diploid wild wheat, *Aegilops tauschii* Coss. (DD genome) [9], [10]. The birthplace of common wheat is considered to lie within the area comprising Transcaucasia and the southern coastal region of the Caspian Sea [11], [12]. Allohexaploid wheat plants can be artificially produced through hybridization of these species and are called synthetic hexaploid wheat [9], [10], [13], [14]. However, abnormal growth phenotypes such as germination failure, hybrid necrosis and hybrid sterility were observed in many F_1_ triploid hybrids (ABD genome) between tetraploid wheat and *Ae*. *tauschii* [15]–[18], which indicated the presence of genetic factors inhibiting the production of common wheat. The hybrid necrosis phenotype in interploidy crosses between tetraploid wheat and *Ae*. *tauschii* was previously subdivided into type II and type I-like. In hybrid plants showing type I-like necrosis, which we defined here as type III necrosis, cell death occurs gradually from older tissues, and similar necrotic cell death was observed in some hybrids between common wheat cultivars [17]. Nishikawa [17] described that type III necrosis was characterized by its occurrence at the second or third leaf stage, anthocyan pigment frequently appeared at the top of leaf blades and gradually spread downward with progression of chlorophyll depletion, and then the plants turned brown and died. On the other hand, type II necrosis lines showed a necrotic phenotype under low temperature conditions [17]. Genealogical analysis based on chloroplast DNA variations revealed that *Ae*. *tauschii* accessions inducing the abnormal growth phenotype in hybrids with tetraploid wheat had a limited geographic and phylogenetic distribution [18]. These observations indicated that postzygotic hybridization barriers are underdeveloped between tetraploid wheat and *Ae*. *tauschii*. The postzygotic barrier occurs due to epistatic interaction between the AB and D genomes, which can be postulated to depend on the BDM model. Although postzygotic barriers between the AB and D genomes might play a significant role in polyploid speciation of common wheat, there is little genetic characterization including chromosome assignment of the causal genes and molecular mechanisms that induce the abnormal growth phenotypes.

Of the hybrid abnormalities, hybrid necrosis is a well-known reproductive isolation associated phenomenon in plant species [2]. A naturally occurring allele of an NB-LRR-type *R* gene was identified as the causal gene of hybrid necrosis in crosses among some combinations of *Arabidopsis thaliana* ecotypes [3]. In this case, necrotic F_1_ plants showed higher transcript accumulation levels of defense-related genes and increased resistance against plant pathogens compared to their parental ecotypes. Elevated levels of proteins and transcripts that are associated with plant pathogenic responses have also been reported in necrotic *Nicotiana* hybrids [19], implying that a hybrid necrosis system genetically induced via a immune response is widely conserved in plant species [2]. In crosses among *Nicotiana* species, F_1_ plants undergoing hybrid necrosis have increased superoxide levels, and chromatin condensation and DNA fragmentation occur, indicating that programmed cell death might be involved in the hybrid necrosis processes [19]. In crosses of common wheat cultivars, *Ne1* and *Ne2* loci are known to control hybrid necrosis [20]. These two complementary genes, *Ne1* and *Ne2*, are located on chromosome arms 5BL and 2BS, respectively [21]–[23]. Wheat plants carrying both *Ne1* and *Ne2* exhibit progressive cell death and abundantly accumulate reactive oxygen species at the seedling stage [24]. The *Ne2* locus shows extremely tight genetic linkage with loci that confer effective rust fungus resistance [2], [25]. The necrotic symptoms in the type I *Ne1*-*Ne2* system closely resemble that of type III necrosis in hybrids between tetraploid wheat and *Ae*. *tauschii* [15], but the causal genes of the type III necrosis are different than those of the type I system because the *Ae*. *tauschii* loci for type III necrosis is included in the D genome but not in the B genome. The relationship between the disease resistance related responses and type III necrosis is still unknown.

Population structure and intraspecific diversification of *Ae*. *tauschii* were well characterized in our previous studies based on chloroplast and nuclear DNA and morphological variations [25]–[28]. Phylogenetic and principal component analyses revealed two major lineages in *Ae. tauschii*. Bayesian population structure analyses based on the AFLP data showed that lineages 1 (L1) and 2 (L2) were significantly divided respectively into six and three sublineages [28]. Only four out of the six L1 sublineages had diverged from those of western habitats in the Transcaucasian and northern Iran regions to eastern habitats such as Pakistan and Afghanistan, whereas the *Ae. tauschii* populations involved in the origin of common wheat have been narrowed down to L2, probably sublineage 2–3 [28]. The first objective of this paper was to elucidate the relationship between hybrid abnormalities and intraspecific differentiation of *Ae*. *tauschii*.

Absence of alleles causing growth abnormalities in triploid hybrids between tetraploid wheat and *Ae. tauschii* is one of the essential factors for successful polyploid speciation of common wheat, similar to the ability to form unreduced gametes and to suppress homoeologous chromosome pairing after hybridization [14], [29]. In addition, hybrid abnormalities act as reproductive barriers inhibiting introgression of agriculturally important alleles from natural populations of *Ae. tauschii* through synthetic hexaploid wheat lines [30]. Therefore, physiological and genetic understanding of those abnormalities occurring in hybrids between tetraploid wheat and *Ae. tauschii* is important for wheat breeding. Of the hybrid abnormalities that occurred in interploidy crosses between tetraploid and wild diploid wheat, type III necrosis was seemingly most similar to other reported hybrid necroses in homoploid crosses based on their phenotypic observations. Accordingly, the second aim of this paper was to elucidate molecular mechanisms of type III necrosis expression at the gene transcriptional and physiological levels. Based on these results, we discus points of commonalities and differences with hybrid necrosis phenotypes reported in other plant cross-combinations.

## Results

### Distribution of the *Ae. tauschii* accessions resulting in abnormal hybrid growth

**Figure 1:**
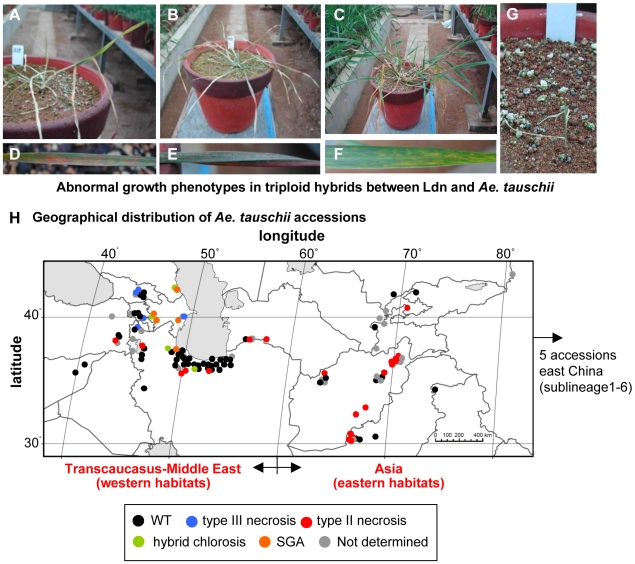
Abnormal growth phenotypes observed in hybrids between tetraploid wheat and *Ae*. *tauschii* accessions. A-C) Overview of type III necrosis (A), type II necrosis (B), and hybrid chlorosis (C) lines at the phase transition stage from vegetative to reproductive growth. D) Type III necrosis symptoms on leaf in (A). E) Type II necrosis symptoms on leaf in (B). F) Hybrid chlorosis symptoms on leaf in (C). G) Overview of severely growth aborted (SGA) line 4 months after sowing. H) Geographical distribution of *Ae. tauschii* accessions showing the four types of abnormal growth phenotypes when crossed into a tetraploid wheat cultivar Langdon.

Synthetic hexaploid wheat can be produced through unreduced gametogenesis in F_1_ hybrid plants crossed between tetraploid wheat cultivar Langdon (Ldn) and various accessions of *Ae. tauschii* [14], [30]. In F_1_ hybrids, however, abnormal growth phenotypes are frequently observed. The abnormal growth phenotypes are divided into four types: two types of hybrid necrosis, hybrid chlorosis, and severe growth abortion (SGA). The two types of hybrid necrosis correspond to types II and III necrosis, which have been reported only in [15]. In triploid hybrids showing type III necrosis, cell death occurred gradually starting from older tissues (Fig. 1A, 1D). On the other hand, hybrid plants showing type II necrosis usually grew until exposed to low temperature, when they clearly exhibited a necrotic phenotype and incomplete leaf sheath emergence at the tillering stage (Fig. 1B, 1E). These two types of hybrid necrosis were not lethal, and selfed seeds could be obtained in spite of the small number of selfed seeds per triploid plant. Leaves of hybrid plants showing chlorosis gradually turned yellowish in an age-dependent manner (Fig. 1C, 1F). Triploid plants with hybrid chlorosis were fertile, and selfed seeds could be obtained. In SGA-exhibiting hybrids, plant growth was severely inhibited and stopped before the third or fourth leaves emerged (Fig. 1G), and thus SGA was lethal to seedlings. In this study, triploid hybrids showing a normal growth phenotype are considered wild type (WT). The number of hybrids formed from *Ae. tauschii* accessions and Ldn showing WT, type III necrosis, type II necrosis, hybrid chlorosis, and SGA were 61 (62.9%), 5 (5.2%), 22 (22.7%), 4 (4.1%) and 5 (5.2%), respectively (Table 1).

**Table 1:** Genealogical distribution of the triploid hybrid phenotypes in sublineages of *Ae*. *tauschii* based on the nuclear DNA variations.

	Sublineage^a^									Lineage^a^	
Hybrid phenotype	1–1	1–2	1–3	1–4	1–5	1–6	2–1	2–2	2–3	1	2	Other	Total
WT		11		3	8	3	8	5	20	25	33	3	61
Type III necrosis		2	2		1					5			5
Type II necrosis	2	14	2	3	1					22			22
Hybrid chlorosis							3		1		4		4
SGA								3	2		5		5
not determined	3	5	2	4	3	2	2	3	1	19	6		25
Total	5	32	6	10	13	5	13	11	24	71	48	3	122

a Mizuno et al. [28].

To study geographical distribution of genetic factors causing the hybrid growth abnormalities in *Ae*. *tauschii*, F_1_ triploid hybrids between Ldn and various accessions of *Ae. tauschii* were obtained and grown. In total, triploid hybrids between Ldn and 97 *Ae. tauschii* accessions (at least two F_1_ seeds per a cross combination) were produced and grown, and their phenotypes were determined and plotted onto the *Ae. tauschii* habitats (Fig. 1H; Table S1; Table S2). There were no triploid hybrids showing two distinct abnormal growth phenotypes. The *Ae. tauschii* accessions inducing either type III necrosis, hybrid chlorosis or SGA in triploid hybrids were found only from western habitats (<60° longitude), whereas the accessions resulting in type II necrosis were mainly from eastern habitats (≥60° longitude, 15/22 = 68.18%). In particular, type III necrosis, chlorosis, and SGA phenotypes were observed only in F_1_ hybrids from the Transcaucasian accessions of *Ae. tauschii*. The accessions resulting in type II necrosis were predominantly found in Afghanistan and Pakistan (65.2%). The accessions producing triploid hybrids without any abnormal growth phenotype (WT) were widely distributed throughout the *Ae. tauschii* habitats, and the east China and Caspian accessions showed high frequencies (100 and 90.6%, respectively) of WT formation.

Our previous study revealed that there are two major lineages, lineages 1 (L1) and 2 (L2), and an HG17 lineage (HGL17) in 122 *Ae*. *tauschii* accessions [28]. Bayesian population structure analyses showed that L1 and L2 are divided into six and three sublineages, respectively. To study the relationship between genetic factors causing the hybrid growth abnormalities and *Ae*. *tauschii* sublineage diversification, phenotypes of F_1_ hybrids between Ldn and the 97 *Ae. tauschii* accessions were plotted onto each sublineage (Table 1). *Ae*. *tauschii* accessions resulting in either of the two necrosis types were observed only in L1, whereas accessions inducing either hybrid chlorosis or SGA were found only in L2. The accessions producing the WT hybrids were distributed in both L1 and L2.

The *Ae*. *tauschii* accessions resulting in type III necrosis, type II necrosis, hybrid chlorosis and SGA were found in restricted sublineages: sublineages 1–2, 1–3 and 1–5 for type III necrosis, sublineages 1–1, 1–2, 1–3, 1–4 and 1–5 for type II necrosis, sublineages 2–1 and 2–3 for hybrid chlorosis, and sublineages 2–2 and 2–3 for SGA (Table 1). The accessions producing the WT hybrids were widely distributed in all but sublineages 1–1 and 1–3. The frequency of accessions resulting in type II necrosis was higher than for other hybrid abnormalities in *Ae*. *tauschii*. Especially in sublineages 1–1, 1–2, 1–3 and 1–4, the frequency of type II necrosis-inducing accessions was 53.8% (21/39). In contrast to type II necrosis, the frequency of type III necrosis-inducing accessions was low in the L1 sublineages. The accessions producing WT hybrids were frequently found in sublineage 2–3.

### Phenotypic characterization of synthetic lines with type III necrosis

**Figure 2:**
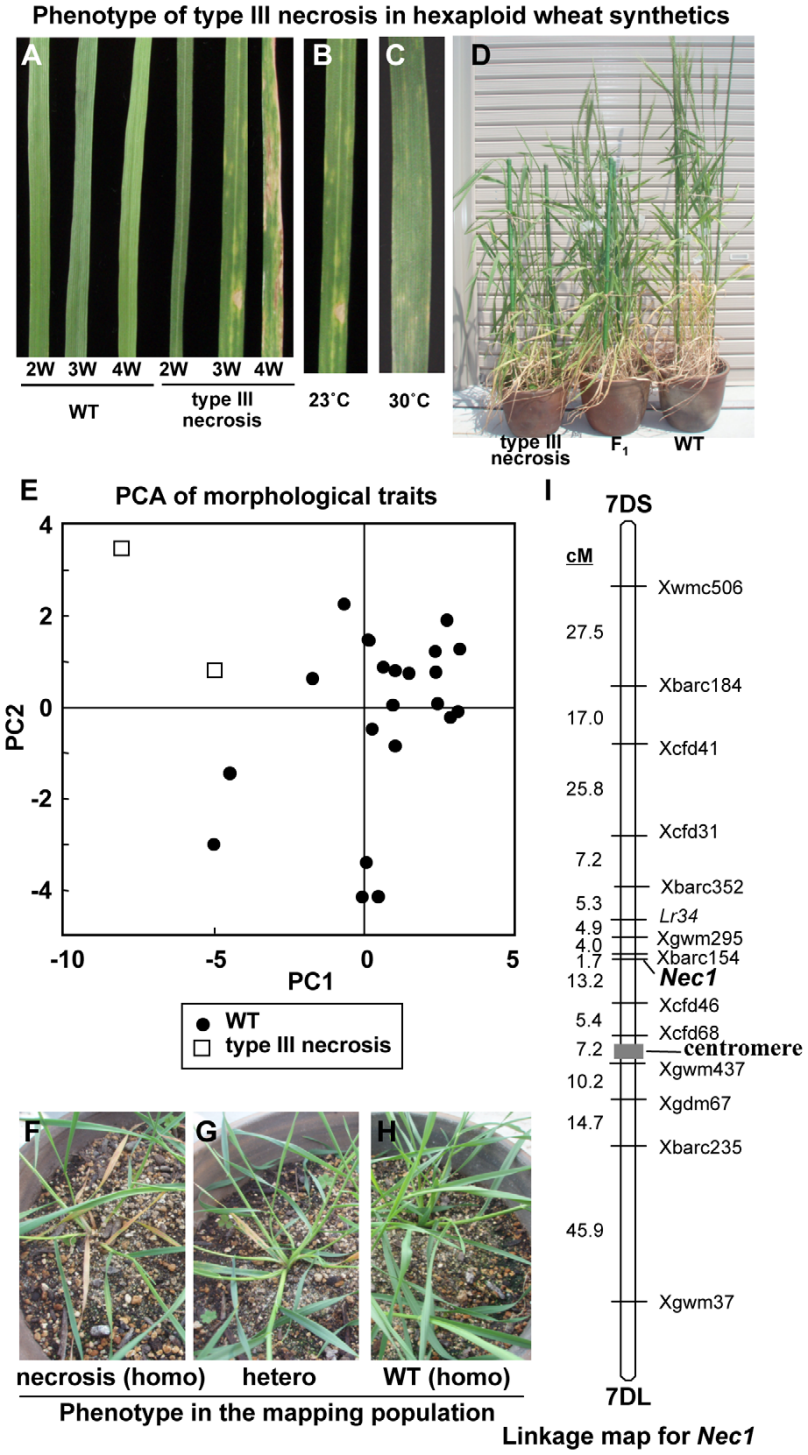
Phenotypic and genetic characterization of type III hybrid necrosis in hexaploid wheat synthetics. A) Phenotype of the 1st (lowest) leaf in WT and a type III necrosis line. From the left, 2-, 3- and 4-week-old seedlings in WT and the type III necrosis line are sequentially shown. Each plant was grown at 23°C. B-C) Leaf phenotype of the type III necrosis line grown at 23°C (B) and 30°C (C). D) Overview of type III necrosis lines. Left; type III necrosis line (synthetic wheat of Ldn and KU-2828), middle; F_1_ plant between the WT and type III necrosis lines, right; WT line (synthetic wheat of Ldn and KU-2075). E) Graph of the first two axes from a principal component analysis based on the 19 morphological traits in synthetic hexaploid wheat lines showing WT and type III necrosis. F) Early growth and leaf phenotype of type III necrosis line (synthetic wheat of Ldn and KU-2828). G) Early growth and leaf phenotype of F_1_ plant between WT and type III necrosis line. H) Early growth and leaf phenotype of the WT line (Ldn x KU-2075). I) Genetic linkage map for *Nec1* on chromosome 7D. Genetic distances (cM) and markers are shown on the left and right, respectively.

Selfed seeds (F_2_ generation; called as synthetic wheat or wheat synthetics) from triploid F_1_ hybrids between Ldn and various *Ae. tauschii* accessions were obtained through unreduced gamete formation. The chromosome number of the synthetic wheat plants was 42 ([30], this study), meaning that the synthetic wheat plants were allohexaploid wheat with the AABBDD genome. In total, five hexaploid wheat lines showing type III necrosis were established from type III necrosis-exhibiting triploid hybrids, each of which was derived from each of 5 different *Ae. tauschii* accessions. Necrotic symptoms appeared on the 1st (lowest) leaves of the type III necrosis lines when the 3rd leaves emerged, and gradually progressed in an age-dependent manner (Fig. 2A). The phenotype of type III necrosis was quite similar to the *Ne1*-*Ne2*-based hybrid necrosis observed in hybrids between common wheat cultivars [24]. It was previously reported that a high growth temperature suppresses necrotic symptoms more effectively than a normal growth temperature in hybrid necrosis of common wheat, *A*. *thaliana* and lettuce [3], [4], [31]. However, the necrotic symptoms of type III necrosis were not alleviated under high temperature conditions (30°C) (Fig. 2B, 2C).

To study the effects of type III necrosis on morphological characters in hexaploid synthetic wheat, a total of 19 morphological characters were measured using 24 synthetic hexaploid wheat lines: two independent lines for type III necrosis and 22 WT lines (Table S3; Fig. 2D). Significant differences were observed in traits related to flowering time, culm length, seed fertility, and seed weight between the WT and type III necrosis lines. Principal component (PC) analysis was performed on the morphological data, and PC1 and PC2 values were calculated for each synthetic line. As a result, PC1 and PC2 of the morphological traits were found to capture 65.5% of the total variation (44.96% for PC1 and 20.53% for PC2). Most traits had positive eigenvectors for PC1, indicating that the variation in PC1 values had a major effect on culm length (Table S4). Variation in PC2 values mainly influenced flowering time and leaf shape. In a graph of PC1 and PC2, WT and type III necrosis lines were positioned separately based on their PC1 and PC2 values (Fig. 2E), indicating that the type III necrosis lines have a distinct morphology from normal wheat synthetics.

### Genetic mapping of the causal gene for type III necrosis

F_1_ plants between WT (synthetic wheat line of Ldn and KU-2075) and type III necrosis (synthetic wheat line of Ldn and KU-2828) hexaploid lines showed lower height than the parental WT line, and exhibited weaker necrotic phenotypes than the parental type III necrosis line. These observations indicated that the F_1_ phenotypes were precisely distinguished from the parental lines and that the necrosis phenotype was affected in a dose-dependent manner of causal alleles of the type III necrosis (Fig. 2D, 2F, 2G, 2H).

To determine the number of causal genes associated with type III necrosis and their chromosome location, an F_2_ mapping population was generated from an F_1_ plant between WT (synthetic wheat line of Ldn and KU-2075) and type III necrosis (synthetic wheat line of Ldn and KU-2828) hexaploid lines. The parental synthetics thus contained the A and B genomes from Ldn and the diverse D genomes originating from the *Ae. tauschii* male parents, meaning that the F_2_ mapping population was useful for genetic analyses of the causal gene on the D genome. In total, 116 F_2_ individuals were segregated into 29 necrosis homozygous, 55 heterozygous, and 32 WT homozygous plants, fitting a 1∶2∶1 segregation ratio (χ^2^ = 0.47, *P* = 0.79). This segregation data indicated that a single genetic locus is associated with type III necrosis on the D genome, and that the D-genome locus complemented a genetic factor on the A or B genome for expression of necrosis symptoms. The D-genome locus for type III necrosis was named *Nec1* (*type III necrosis 1*).

To assign the *Nec1* locus to the D-genome chromosome, linkage analysis using 39 simple sequence repeat (SSR) markers located on each chromosome arm of the D genome was conducted in the F_2_ mapping population, and *Nec1* was linked to chromosome 7D. Next, genetic mapping using 13 polymorphic SSR markers on chromosome 7D showed that *Nec1* could be assigned to the short arm of chromosome 7D and that *Xbarc154* was closely linked with *Nec1* (1.7 cM) (Fig. 2I). In common wheat, the leaf rust-resistance gene *Lr34* on chromosome arm 7DS was recently isolated by a map-based approach [32]. To map the *Lr34* locus on the 7DS linkage map, a 5-bp indel polymorphism in the 4th intron of *Lr34* was used as a marker. As a result, *Lr34* was located 10.6 cM distal from *Nec1* (Fig. 2I).

### Alteration of transcriptome in type III necrosis

**Figure 3:**
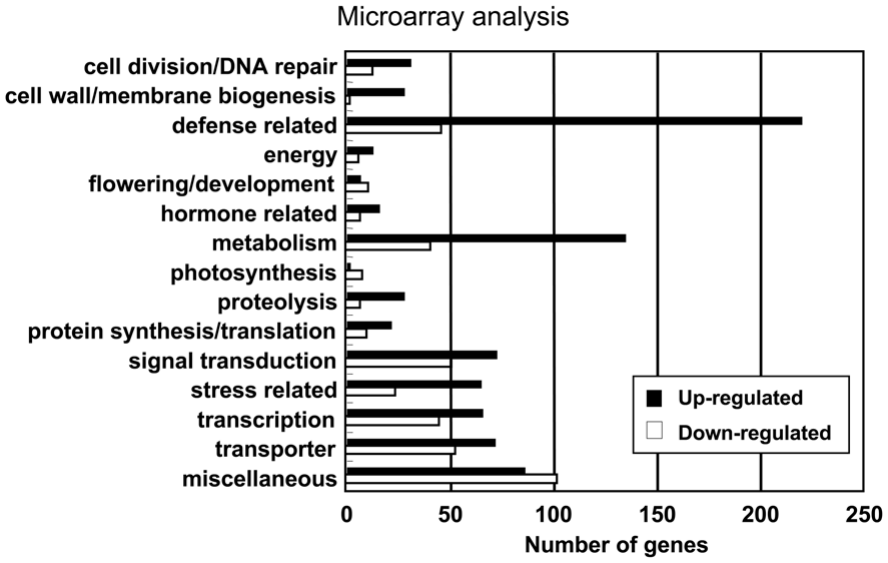
Summary of microarray analysis. Up- and down-regulated genes in type III necrosis were categorized into fifteen functional groups.

To compare comprehensive gene expression patterns between the two synthetic hexaploid lines from crosses of Ldn and KU-2059 as a WT line and of Ldn and KU-2828 as a type III necrosis line, transcriptome analysis was performed using a wheat 38K oligo-DNA microarray [33]. After hybridization with total RNA samples from 3-week-old seedling leaves, probes showing at least a 3-fold difference in signal intensity compared to WT were defined as up- or down-regulated genes. Out of the 37,826 probes on the wheat microarray, 1,521 (4.0%) and 965 (2.6%) probes were up- and down-regulated in the type III necrosis line, respectively. Based on homology of these probes in searches of the wheat EST database, 864 (56.8%) of the up-regulated and 423 (43.8%) of the down-regulated genes were categorized into 15 groups based on their inferred functions. Gene functions of the remaining probes are unknown. Of the up-regulated genes, defense-related genes were most frequently (25.6%) found in the 15 groups (Fig. 3; Table S5). Metabolism (15.6%), signal transduction (8.4%) and transport related groups (8.3%) in addition to transcription factors (7.6%) were also abundantly expressed in the type III necrosis line. On the other hand, transport (12.5%), defense (10.9%), transcription (10.4%), metabolism (9.9%) and stress related groups (5.7%) were down-regulated in the type III necrosis line.

Among the defense related genes, pathogenesis related (PR) genes and flavonoid biosynthesis related genes such as phenylalanine ammonia-lyase (PAL) and chalcone synthase (CHS) genes were highly up-regulated in the type III necrosis line (Table S5). These flavonoid biosynthesis related proteins including PAL and CHS function in biosynthesis of plant natural products based on the phenylpropane skeleton such as lignin monomers and phytoalexins [34]. In addition, chitinase, lipoxygenase, peroxidase and glutathione S-transferase encoding gene transcripts accumulated abundantly in the type III necrosis line. Alternative oxidase (AOX) and catalase genes, which function in alleviation of reactive oxygen species (ROS) generation, were up-regulated in microarray analysis (Table S6). Fourteen transcription factor genes belonging to the WRKY family were also up-regulated. Thus, the up-regulation of a number of defense related genes was characteristic of the type III necrosis line.

On the other hand, photosynthesis related gene groups showed the highest rate of down-regulated genes to the up-regulated genes in the 15 functional groups (Fig. 3). Many photosynthesis related genes such as *psbB*, *psbE* and *ndhB* were down-regulated in the type III necrosis line (Table S6). These results indicated that expression of photosynthesis related genes was mainly suppressed in type III necrosis-exhibiting plants.

**Figure 4:**
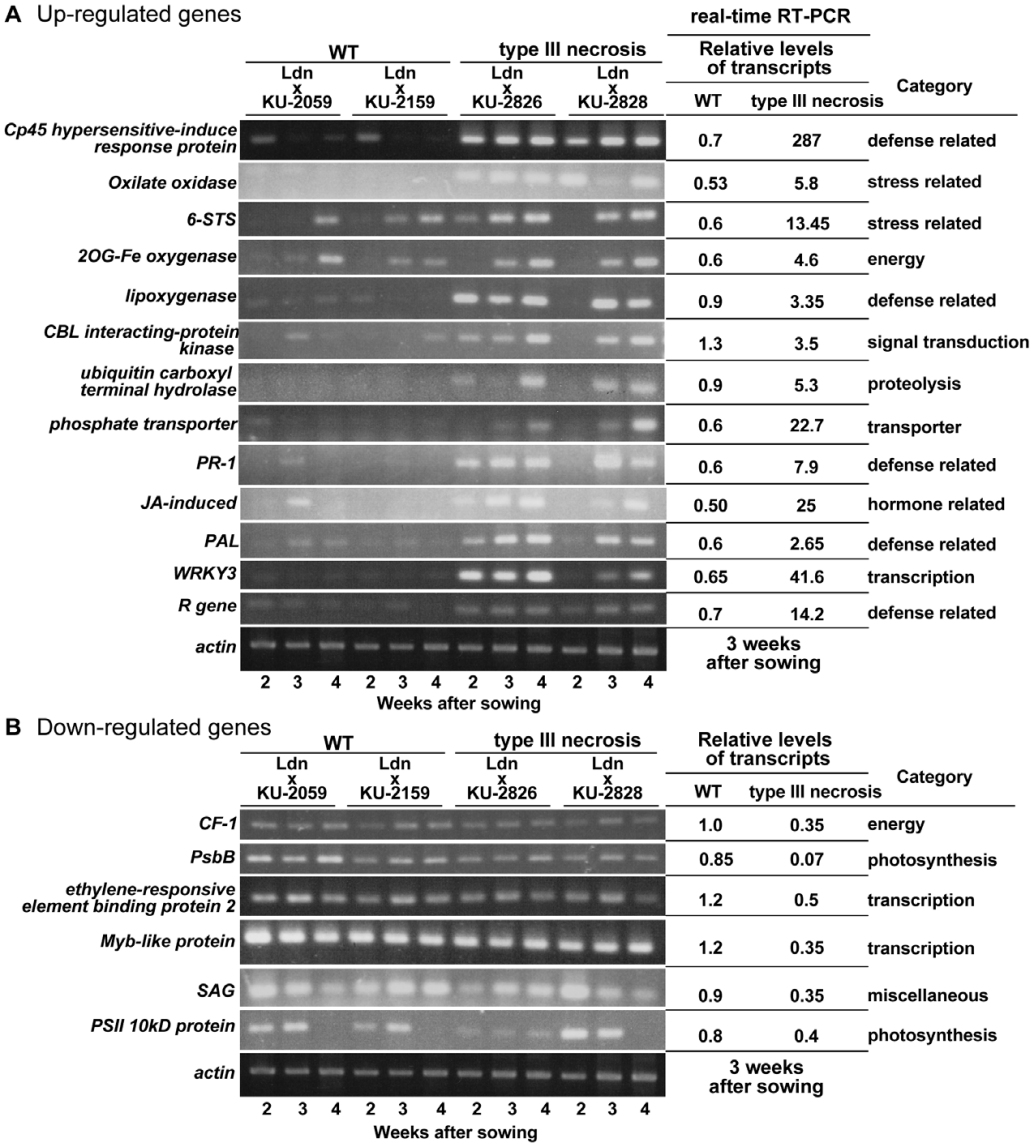
Alteration of transcript accumulation levels in type III necrosis. RT-PCR and real-time RT-PCR analyses were performed using probe-specific primers. Total RNA was extracted from the 1st leaves of 2-, 3- and 4-week-old seedlings. The *Actin* gene was used as an internal control. Mean of relative transcript levels of the WT and type III necrosis lines revealed by real-time RT-PCR analysis is represented on the right side of the electropherograms. Values were calculated relative to the transcript levels in 3-week-old seedling leaves of the WT line (synthetic wheat of Ldn and KU-2059). A) Expression profiles of up-regulated genes identified by microarray analysis. B) Expression profiles of down-regulated genes identified by microarray analysis.

To validate the microarray data, RT-PCR and quantitative RT-PCR analyses for 22 selected genes were performed using the two synthetic wheat lines used in the microarray analysis and two additional synthetic lines; one was a WT line from a hybrid of Ldn and KU-2159 another a type III necrosis line obtained from crossing Ldn and KU-2826. Total RNA samples were extracted from 2-, 3-, and 4-week-old seedling leaves for a time-course study. Of the examined 15 up-regulated and 7 down-regulated genes from the 10 gene categories, the expression pattern of only one down-regulated gene was inconsistent with the microarray data. In the other 21 genes (95.5%), differences in transcript accumulation levels between the 3-week-old leaves of the two synthetic lines used in the microarray analysis corresponded to the microarray results (Fig. 4). However, comparison of the 21 gene expression patterns among the four wheat synthetic lines indicated that differences in two up-regulated genes were independent of the phenotypic difference. Finally, differences in the expression patterns observed in the 13 (86.7%) up-regulated and 6 (85.7%) down-regulated genes were consistent with both the microarray results and the phenotypic difference between WT and type III necrosis. Therefore, the RT-PCR analyses generally showed similar expression patterns to those observed by microarray analysis.

### Cytological evaluation and ROS detection in leaf cells showing type III necrosis

**Figure 5:**
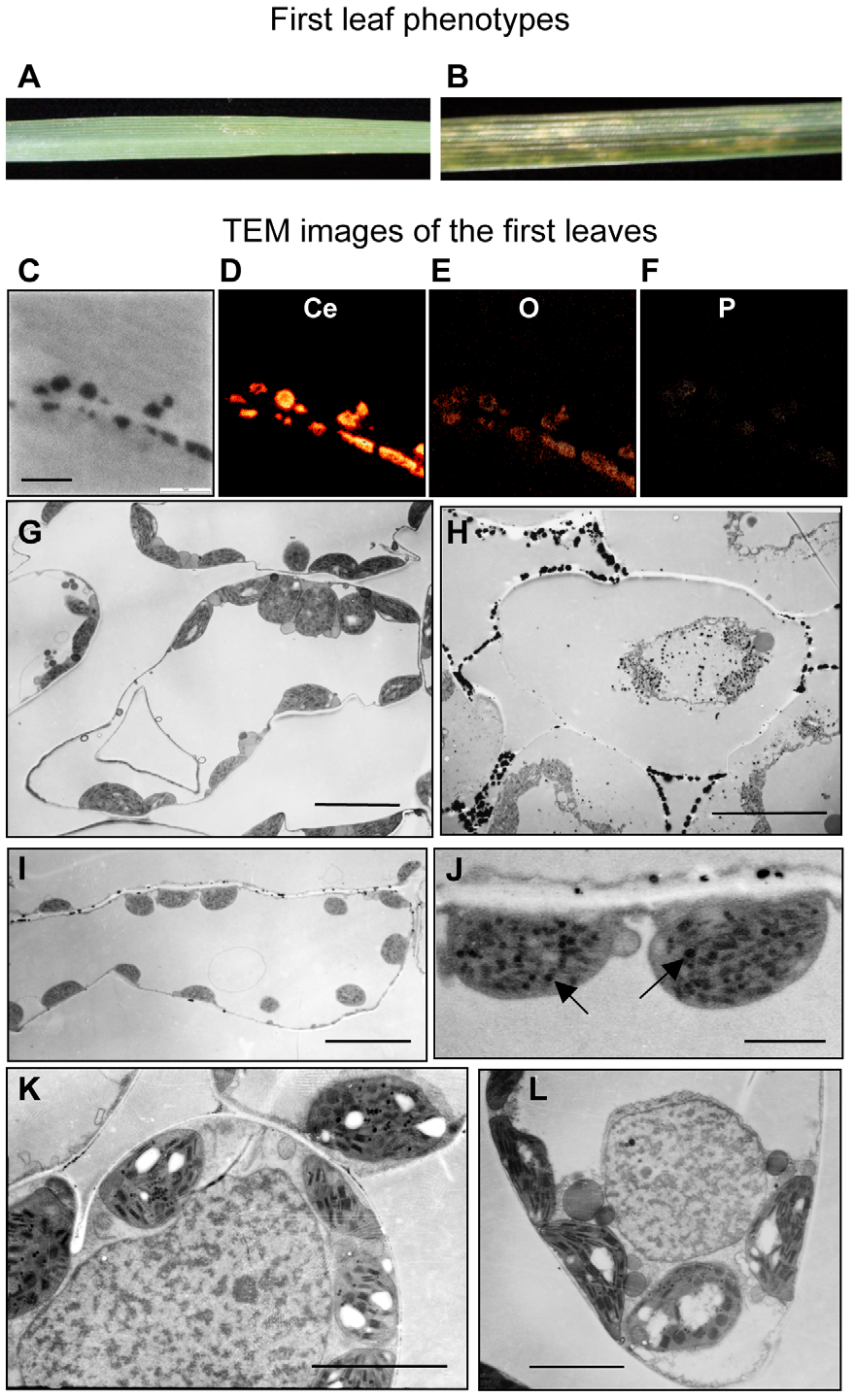
Transmission electron microscopic (TEM) observations of the intracellular structure of the 1st (lowest) leaves in the synthetic hexaploid wheat lines. Each plant was grown at 23°C for 3 weeks, and the leaves were pre-treated with CeCl_3_ for ROS detection. A–B) The 1st leaf for TEM observation in wild type (A) and type III necrosis line (B). C–F) Elemental mapping using energy-filtered transmission electron microscopy. Dark cerium-reacting deposits were observed in TEM analysis (C). Each orange fluorescence indicates localization of cerium, oxygen and phosphorus in (D), (E) and (F), respectively. The bar in (C) indicates 1 µm. G-L) Mesophyll cells in the 1st leaves of the WT (G, L) and type III necrosis (H, I, J, K) lines. Arrows in (J) indicate oil droplets. Bars indicate 10 µm (G, H and I), 5 µm (K and L) and 2 µm (J).

ROS generation has been observed in wheat and *Nicotiana* plants undergoing hybrid necrosis [19], [24]. In our microarray analysis of type III necrosis, AOX and catalase genes functioning in ROS alleviation were found to be up-regulated (Table S6). To study in detail the cytological and physiological changes in type III necrosis, intracellular structures of mesophyll cells were compared between the two synthetic hexaploid lines from crosses of Ldn and KU-2059 (WT) and of Ldn and KU-2828 (type III necrosis) by transmission electron microscope (TEM) observation. Necrotic symptoms were observed on the 1st (lowest) leaves of 3-week-old seedlings in the type III necrosis line; at the same stage, no necrotic symptoms were found on the 2nd leaves in the type III necrosis line or on the 1st and 2nd leaves of the WT line (Fig. 5A, 5B). For ROS detection under TEM observation, CeCl_3_ was added to the fixation solution. Cerium pre-treatment is a highly sensitive procedure to localize intracellular H_2_O_2_, and ROS detection is based on reaction of H_2_O_2_ with CeCl_3_ to produce insoluble precipitates of highly electron-dense cerium perhydroxides [35]. Therefore, big black deposits representing the insoluble precipitates of cerium perhydroxides should be observed by TEM when H_2_O_2_ has formed in mesophyll cells. To validate the location of H_2_O_2_ generation sites in mesophyll cells by the use of CeCl_3_, elemental mapping was performed using an energy-filtered transmission electron microscope because cerium ions reacting with endogenous PO_4_^2-^ form cerium phosphates [36]. The cerium signals completely corresponded to the black deposits within the intercellular space in type III necrosis (Fig. 5C, 5D). The cerium-reactive deposits also included oxygen but not phosphorus (Fig. 5E, 5F). These results indicated that the CeCl_3_-treatment method functioned well for ROS detection in wheat mesophyll cells.

Plasmolysis, breakage of the cell membrane, collapse of the vacuole, and organelle degradation were the typical features in dead cells. Small cells were recognized as dead in the WT line and the 2nd leaf of the type III necrosis line (Fig. 5G). In mesophyll cells of the 1st leaves in the type III necrosis line, a number of different dead cells were clearly observed (Fig. 5H). In addition, a number of cerium deposits were detected not only in the intercellular space around the dead cells but also in disrupted organelles of the dead cells in the type III necrosis line (Fig. 5H), whereas the WT lines and 2nd leaves of the type III necrosis line showed few deposits (Fig. 5G). These observations indicated that a large amount of H_2_O_2_ was generated in the necrosis-exhibiting leaves. H_2_O_2_ generation was also detected in the intercellular space around living cells adjacent to the dead cells in the 1st leaves of the type III necrosis line, although the cerium perhydroxide deposits were fewer and smaller in the living than the dead cells (Fig. 5I). This indicated that ROS generation occurred prior to cell death. A lot of fat droplets were also found within chloroplasts of living cells in the 1st leaves of the type III necrosis line (Fig. 5J, 5K). Within chloroplasts of the WT line, the number of fat droplets was smaller, and granum-lamella structures were well developed (Fig. 5L). The granum-lamella structure was abnormal and linear formation of grana was not observed within the chloroplasts of the living cells in the type III necrosis line (Fig. 5J, 5K), implying that these chloroplasts were in a state of disruption. On the other hand, no significant difference in chromatin condensation was observed between the WT and type III necrosis lines (Fig. 5K, 5L).

**Figure 6:**
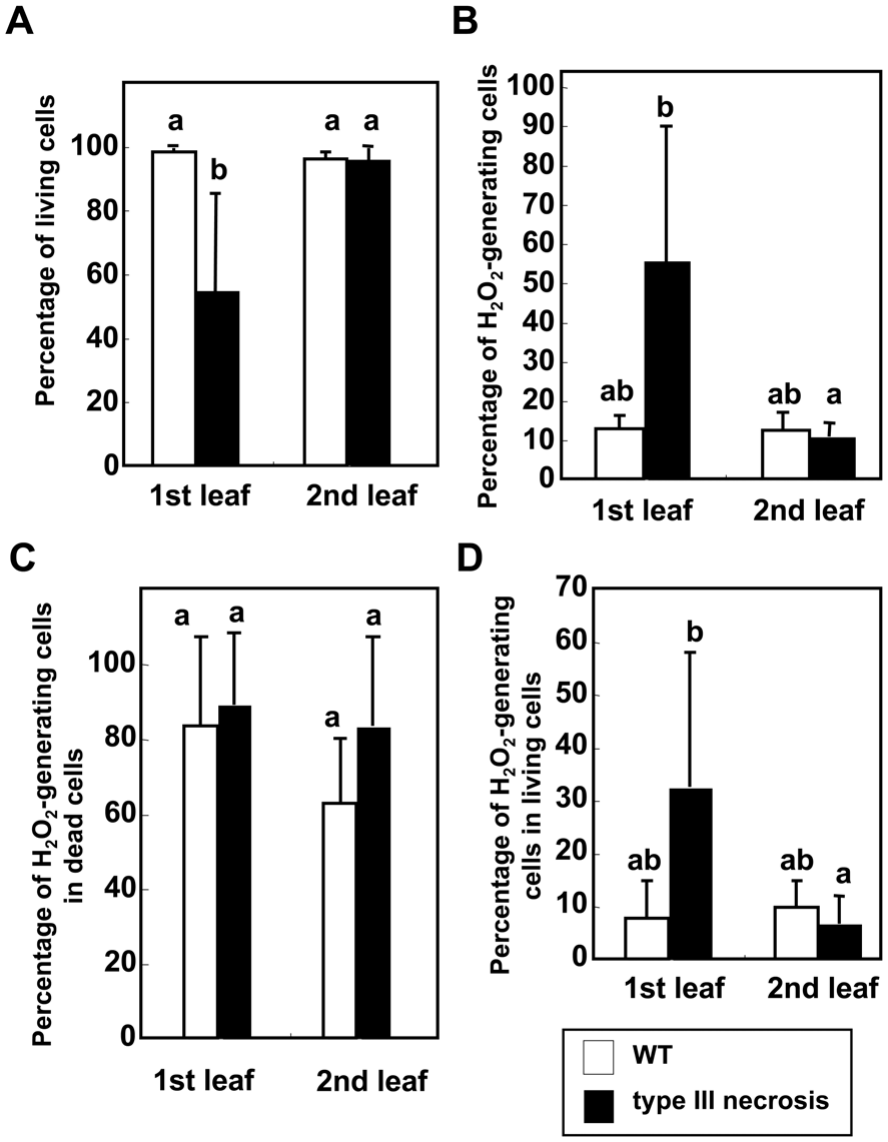
Relationship between cell death and ROS generation revealed by TEM observations. Mesophyll cells of the 1st (lowest) and 2nd leaves were compared for synthetic hexaploid wheat lines showing WT and type III necrosis. Means ± standard errors were calculated from data in three experiments. Mean values with the same letters were not significantly different (*P*>0.05) (Turkey-Kramer's HSD test). A) Percentage of observed living mesophyll cells. B) Percentage of observed mesophyll cells with ROS generation. C) Percentage of dead cells with ROS generation. D) Percentage of living cells with ROS generation.

The frequency of living cells was significantly lower in the 1st leaves of the type III necrosis line than in the WT line or the 2nd leaves of the type III necrosis line (Fig. 6A). In the 1st leaves of the type III necrosis line, the frequency of cells with H_2_O_2_ was significantly higher than in the other leaf samples (Fig. 6B). Most dead cells showed ROS generation in each leaf sample, and no significant difference in the frequency of dead cells with ROS was observed between leaf samples of the WT and type III necrosis lines (Fig. 6C). On the other hand, the frequency of living cells with ROS was significantly higher than in the WT line or the 2nd leaves of the type III necrosis line (Fig. 6D). Significantly more living cells generating ROS were in the wheat synthetics undergoing type III necrosis than in normally growing synthetics.

**Figure 7:**
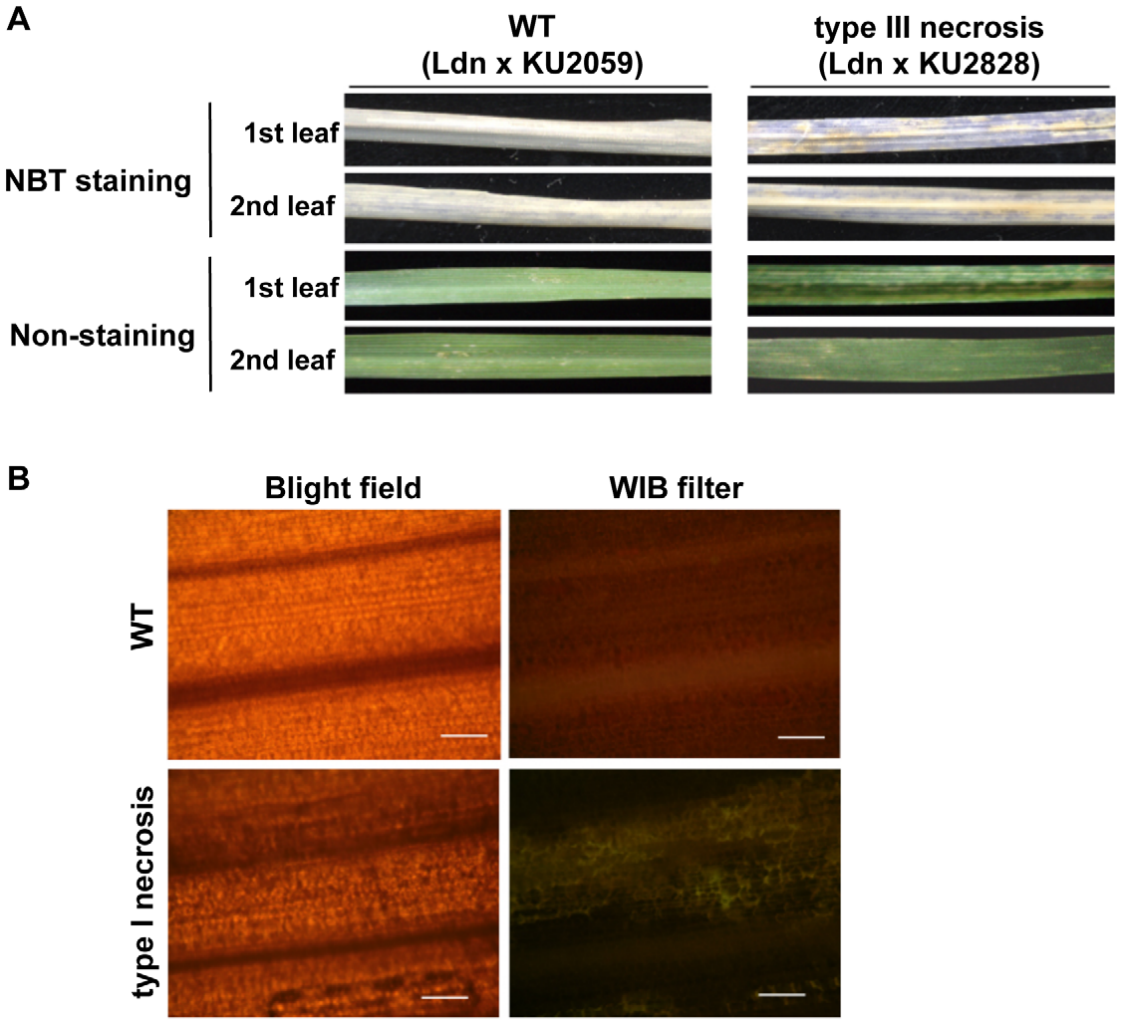
Physiological differences between WT and type III necrosis. A) ROS generation in the 1st and 2nd leaves of the WT and type III necrosis lines revealed by NBT infiltration assay. Each plant was grown at 23°C for 3 weeks before staining. B) Mimicking of the hypersensitive response by type III necrosis. Yellow autofluorescence was detected only in type III necrosis lines. Fluorescence micrographs are shown in the right panels, and bright field images are in the left panels. Bars indicate 100 µm.

**Figure 8:**
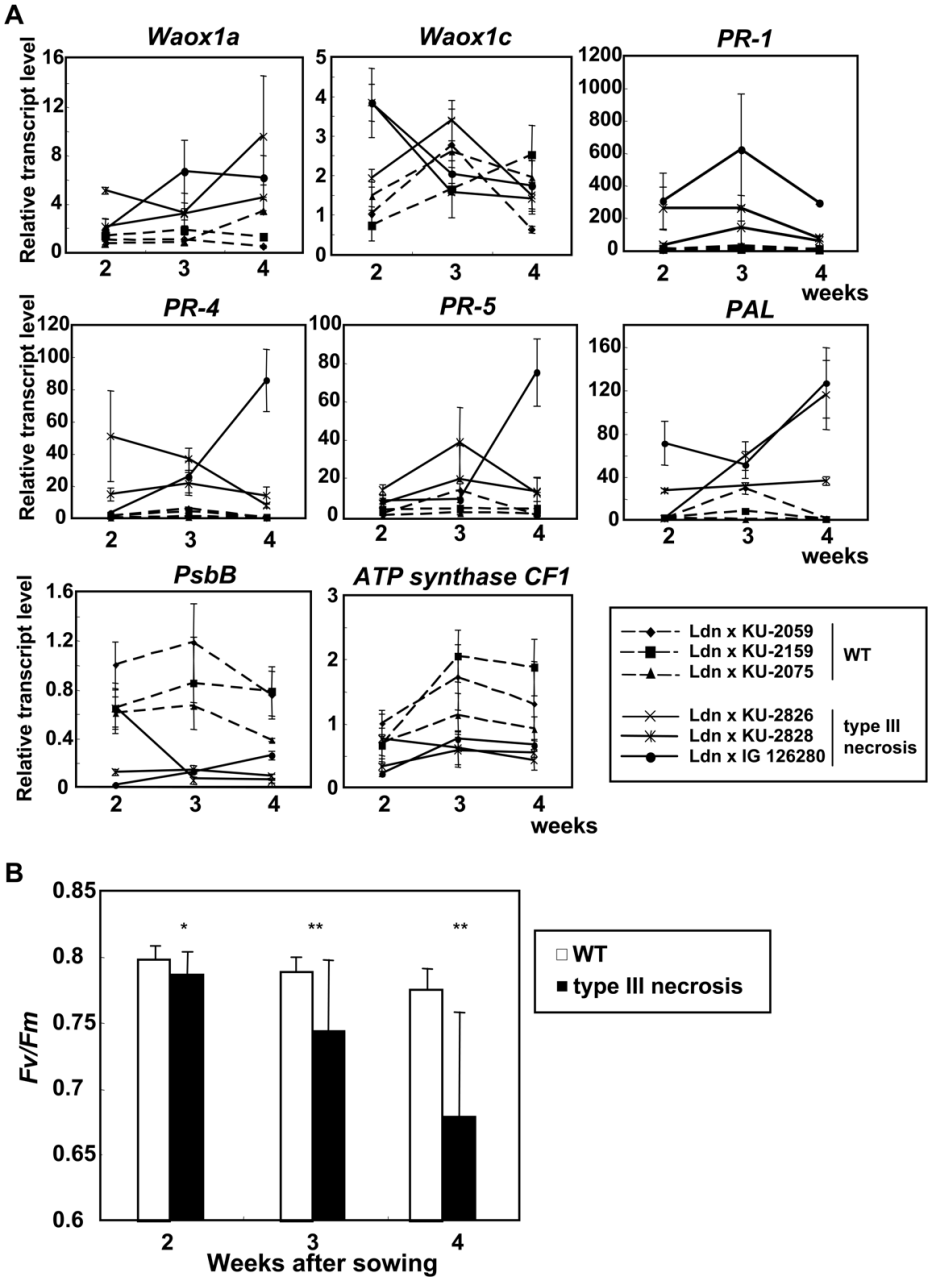
Transcript accumulation and photosynthetic activity in WT and type III necrosis. A) Quantitative RT-PCR of two alternative oxidase genes, four defense related genes, and two photosynthesis related genes. Total RNA was extracted from the 1st leaves of 2-, 3- and 4-week-old seedlings. The transcript levels are shown as values relative to those in 2-week-old seedlings of the WT line (synthetic wheat of Ldn and KU-2059). Means ± standard deviations were calculated from data in three experiments. The *Actin* gene was used as an internal control. B) Reduction of photosynthetic activity in type III necrosis. Chlorophyll fluorescence was compared between the WT and type III necrosis lines. The 1st leaves of 2-, 3- and 4-weeks-old seedlings were incubated in the dark for 2 h and then used to calculate the ratio of the variable to maximum fluorescence (*Fv/Fm*). Means ± standard deviations were calculated from data in ten experiments. Student's *t*-test was used to test for statistical significance (*P<0.05, **P<0.01) between the WT and type III necrosis lines.

Nitroblue tetrazolium chloride (NBT) is widely known as an O_2_^−^ indicator. NBT staining was also performed using whole leaf blades to compare ROS generation between the WT and type III necrosis lines that were used for TEM observation (Fig. 7A). This histochemical staining assay showed that O_2_^−^ accumulated more abundantly in the 1st leaves than in the 2nd in the type III necrosis line, and little O_2_^−^ was observed in the 1st and 2nd leaves of the WT line. Transcript accumulation levels of *Waox1a* increased until at least 4 weeks after sowing in the three type III necrosis lines, whereas no obvious changes were observed in those of the WT lines (Fig. 8A). No significant differences were detected between WT and type III necrosis lines in *Waox1c* transcript levels. These results indicated that *Waox1a* expression was up-regulated during progression of necrotic symptoms in type III necrosis similarly to type I hybrid necrosis.

### Physiological and molecular evaluation of photosynthetic activity and autoimmune response in type III necrosis lines

The hypersensitive response (HR) is a typical reaction in expression of disease resistance against plant pathogens in plants. To examine the relationship between HR and type III necrosis, the yellow autofluorescence of leaf cells was monitored and compared for the WT and type III necrosis lines under a fluorescent microscope. Yellow autofluorescence was clearly observed in a number of cells in leaves undergoing type III necrosis, whereas it was rarely detected in the WT leaves (Fig. 7B). To examine the defense response in type III necrosis in detail, quantitative RT-PCR analysis was performed using four defense related genes as markers, and leaf age-dependent expression patterns were compared among the three WT and three type III necrosis lines. Transcripts of three pathogenesis related (PR) protein encoding genes, *PR-1*, *PR-4* and *PR-5*, accumulated abundantly in the three type III necrosis lines implied that this is relative to WT (Fig. 8A). PAL is one of the key enzymes of phytoalexin biosynthesis and is highly induced on infection by pathogens [34]. Accumulation levels of the *PAL* transcripts increased as the necrotic symptoms progressed (Fig. 8A).

To validate the down-regulation of photosynthesis related genes, quantitative RT-PCR analysis of the *PsbB* and *ATP synthase CF-1* genes was performed, and leaf age-dependent expression patterns were compared among the three WT and three type III necrosis synthetic lines. PsbB, the 47-kD chlorophyll-a binding protein, is a photosystem II (PSII) component, and CF-1 ATP synthase is integrated into the thylakoid membrane and functions in the Calvin cycle [37], [38]. In the three type III necrosis lines, both *PsbB* and *ATP synthase CF-1* transcripts accumulated less throughout the examined period than in the three WT lines (Fig. 8A). To examine the effects of down-regulation on photosynthetic activity, the photosynthetic activity was compared for synthetic hexaploid lines from crosses of Ldn and KU-2059 (WT) and Ldn and KU-2828 (type III necrosis). The PSII activity of the type III necrosis line was significantly reduced in the 1st leaves of 2-, 3-, and 4-week-old seedlings compared to the WT lines, and the reduction became larger as necrotic symptoms progressed (Fig. 8B). The down-regulation of the photosynthesis related genes was consistent with the reduction in photosynthetic activity during type III necrosis, and the reduction occurred prior to the appearance of necrotic symptoms on the leaves exhibiting type III necrosis.

## Discussion

### Various genetic factors result in wheat hybrid abnormalities

The large-scale production of *Triticum*-*Aegilops* hybrid plants led us to understand the various abnormal phenotypes that occur in triploid hybrids between the AB genomes and the D genome, being the four typical hybrid abnormalities: two types of hybrid necrosis, hybrid chlorosis and SGA (Fig. 1). In this study, the A and B genomes of the triploid hybrids were derived only from *T. turgidum* Ldn, and other tetraploid wheat accessions were previously implicated in abnormal phenotypes [15]. Among the phenotypes reported, abnormal phenotypes similar to type II necrosis, hybrid chlorosis and SGA were recognized, whereas dwarf phenotypes with excess tillers missing in F_1_ hybrids between Ldn and *Ae*. *tauschii*. These various postzygotic hybridization barriers should be considered genetic factors inhibiting the polyploid speciation of common wheat. Therefore, only *Ae*. *tauschii* accessions without such inhibiting factors are candidates for the past D-genome donor for common wheat.

The postzygotic barriers between the AB and D genomes could be controlled by genetic interactions as predicted by the BDM model. Genetic analysis demonstrated that the causal gene inducing type III necrosis on the D genome acted in a semi-dominant manner, and could be assigned to chromosome 7DS (Fig. 2). The phenotype of the type III necrosis visually resembled that of the *Ne1*-*Ne2*-type hybrid necrosis. However, necrotic symptoms were expressed under both normal and high growth temperature conditions in type III necrosis (Figs. 2B, 2C). Nishikawa [16], [17] postulated that expression of type III necrosis is under the control of the *Ne1* and *Ne2* tetraploid wheat genes and the *Ne3 Ae. tauschii* gene, but in the present wheat gene nomenclature the *Ne2* and *Ne3* genes have respectively been revised to the *Ch1* and *Ch2* hybrid chlorosis genes assigned to chromosomes 2A and 3D in common wheat, respectively [39], [40]. Causal genes of the *Ne1*-*Ne2*-type hybrid necrosis have been assigned to chromosomes 5B and 2B [21], [22], and it has been reported that Ldn is a *Ne1* carrier on chromosome 5B [22]. Therefore, the causal gene inducing type III necrosis on chromosome 7DS did not correspond to any previously reported genes causing hybrid necrosis in wheat, and thus we have named the 7DS gene *Nec1*. At least five accessions caused type III necrosis in triploid hybrids with Ldn, and no accession differences were observed for the necrotic symptoms. Together with the observation of their restricted distribution in western habitats, these five *Ae. tauschii* accessions might share an identical allele of the *Nec1* locus. On the other hand, there was no information about chromosomal location of a complementary gene to *Nec1* in tetraploid wheat. *Ae. tauschii* has developed various postzygotic hybridization barriers against the AB genomes, and the genetic bases underlying the hybridization barriers are distinct from those inducing barriers in hybrids among common wheat varieties. Further studies are needed to identify other causal genes for hybridization barriers between the AB and D genomes.

Geographic and genealogical analyses of the distribution of the *Ae. tauschii* accessions inducing the four types of abnormal growth phenotypes in triploid hybrids with Ldn showed that the hybrid abnormality causing accessions have limited geographic and phylogenetic distribution (Fig. 1; Tables 1, S2). The species' putative primary region of origin is Transcaucasia, where diploid species of the genus *Aegilops* radiated 2.5–4.5 million years ago [12]. Therefore, the eastern accessions dispersed from the western habitats [25]. The *Ae. tauschii* accessions inducing type II necrosis were limitedly distributed in L1, of which four sublineages 1–1, 1–2, 1–4 and 1–6 contributed to the west-to-east dispersal of *Ae. tauschii* [28]. The causal gene for type II necrosis in *Ae. tauschii* might develop in the western habitats, and then dispersed into the eastern habitats together with the west-to-east dispersal of the L1 accessions. On the other hand, fewer accessions induced type III necrosis, hybrid chlorosis and SGA than type II necrosis. These postzygotic hybridization barriers developed independently in both major lineages after L1 and L2 diverged. Generation of type III necrosis was a later event in L1 than that of type II necrosis. The three postzygotic hybridization barriers of type III necrosis, hybrid chlorosis and SGA might have developed locally in Transcaucasia.

For the birth of common wheat in the area comprising Transcaucasia and the southern coastal region of the Caspian Sea, Dvorak et al. [12] suggested that the D genome of hexaploid wheat is a composite of several sources, and that subspecies *strangulata* is a possible major D-genome donor. Our previous study showed that the *strangulata* accessions belong to L2 and most are found to a limited extent in sublineage 2–3, and therefore suggested that the D-genome donor could at least be classified to L2 and probably to sublineage 2–3 [28]. In sublineage 2–3, the frequency of *Ae*. *tauschii* accessions causing abnormal growth phenotypes in triploid hybrids with Ldn was significantly lower than in other sublineages (Table 1), supporting the hypothesis that the D-genome donor for common wheat belongs to sublineage 2–3. Therefore, genetic factors causing hybrid abnormalities might at least partly result in the genealogical bias and narrow distribution range of the *Ae. tauschii* populations that involved in the origin of common wheat.

### HR-like cell death occurred in type III necrosis

In intraspecific hybrids of *Arabidopsis*, an NB-LRR-type *R* gene homolog from one parent interacts with an allele at a single locus from a second strain, and the epistatic interaction between these genes is suggested to trigger an autoimmune-like response [3]. Transcriptome analysis showed significant overrepresented categories associated with the plant defense response to pathogen infection in necrotic symptom-exhibiting *Arabidopsis* hybrids. Intraspecific hybrid necrosis in *Nicotiana* has also been linked to the activation of genes associated with defense responses [19], [41]. Our comparative transcriptome analysis in this study clearly indicated that a number of defense related genes were up-regulated in hybrids undergoing type III necrosis (Fig. 3; Table S5). Similarly to *Arabidopsis* intraspecific and *Nicotiana* interspecific hybrids, an autoimmune response might be triggered by intergenomic incompatibility between the AB and D genomes in type III necrosis. Type III necrosis was equally observed in triploid hybrids and in hexaploid wheat synthetics, indicating that the necrotic phenotype was not affected by ploidy. Type I necrosis has been observed in pentaploid hybrids between tetraploid wheat and common wheat [20], [22]. Therefore, autoimmune response inducing hybrid necrosis occurs in polyploid speciation as well as homoploid speciation.

Hypersensitive cell death occurs as a plant defense response against various pathogens [42]. Hypersensitive response (HR) is partly characterized by ROS generation, chromatin condensation, chloroplast disruption, autofluorescence, and activation of PR proteins [42]. TEM observations in leaf mesophyll cells showed that H_2_O_2_ accumulated abundantly in the intercellular space around living cells as well as dead cells of leaves undergoing type III necrosis, and the H_2_O_2_ generation occurred prior to cell death in type III necrosis (Figs. 5, 6). An oxidative burst occurs in the early stage of defense responses to pathogen infection [43], suggesting that the generated ROS may function as initiators of cell death in type III necrosis as they do in HR. Wheat mitochondrial AOX is considered to function in OS alleviation processes under low temperature conditions and in the *Ne1*-*Ne2*-type (type I) hybrid necrosis [24], [44]. Two AOX cDNA molecules, *Waox1a* and *Waox1c*, were previously identified in common wheat [45], and transcript accumulation levels of *Waox1a* were more sensitive to necrotic symptoms than those of *Waox1c* [24]. The up-regulation of *Waox1a* also supported the ROS generation is accompanied by type III necrosis (Fig. 8). It is well known that ROS generation can activate expression of PR proteins and accumulation of phytoalexins [46]. In fact, our expression analyses showed that a large number of defense-related transcripts including genes involved in the phytoalexin biosynthetic pathway were up-regulated (Figs. 3, 8). The transcriptome profile of type III necrosis was very similar to those of *Arabidopsis* intraspecific hybrids showing hybrid necrosis [3].

In HR-inducing cells, various hydrolytic enzymes are released into the cytoplasm due to collapse of the central vacuole, and thus yellow autofluorescence can be detected due to polymerization of phenolics on illumination with blue light [47]. In a previous report on the interspecific hybrid necrosis of *Nicotiana* species, autofluorescence was used as a marker for HR-like cell death [19]. In addition, yellow autofluorescence was specifically observed in leaves undergoing type III necrosis (Fig. 7). Localization of autofluorescence did not always correspond to the visible necrotic regions, inferring that autofluorescence occurred prior to appearance of the necrotic symptoms on leaves exhibiting type III necrosis. Based on TEM observations, even in living cells, the granum-lamella structure was abnormal and linear formation of grana did not occur within the chloroplasts (Fig. 5). These results indicated that the cell death occurring in type III necrosis corresponds well to the HR cell death of plant defense responses. Although no significant difference in chromatin condensation of the WT and type III necrosis lines were observed in living cells compared by TEM (Fig. 5), the genetically programmed cell death in type III necrosis triggered by intergenomic incompatibility could be regarded as an HR-like cell death. The ROS generation in type III necrosis might contribute to the initiation of cell death similarly to HR.

In type III necrosis lines, photosynthetic activity and photosynthesis related gene expression levels significantly decreased as necrotic symptoms progressed compared to WT (Fig. 8). A previous study on bacterially elicited HR in *Arabidopsis* showed a reduction in chlorophyll fluorescence, which is indicative of PSII damage [48]. The severe damage of chloroplasts and reduction in photosynthetic activity might be connected with alteration of various agronomical and morphological traits in the type necrosis lines (Table S3; Fig. 2). Our empirical study showed that values for sink organ related traits such as seed weight and selfed seed fertility were more severely decreased than source organ related traits (Table S3). Selfed progeny can be produced in the type III necrosis lines. However, due to the negative effects on agronomical traits, synthetic wheat lines expressing type III necrosis are not available for wheat breeding. If any agriculturally important genes are identified in *Ae. tauschii* accessions inducing hybrid necrosis, recombinants without *Nec1* should be isolated from crossed progeny with the phenotypically WT accessions before F_1_ triploid hybrid formation with tetraploid wheat.

In this study, we showed that the autoimmune species response occurred in interspecific hybrids of the *Triticum*-*Aegilops* through the epistatic interaction between the different genomes. Several causal genes for hybrid necrosis have been isolated in *Arabidopsis* and lettuce, and most of them encode the NB-LRR-type *R* gene or R-interacting protein gene [3], [4], implying that the autoimmune response via epistatic interaction of *R* genes is a common mechanism of hybrid necrosis in plant species. Rapidly evolving gene families such as NB-LRR and F-box proteins are involved in sensing external inputs including pathogens and can similarly trigger programmed cell death [5], and the rapid evolutionary feature of the *R* genes is necessary to mediate hybrid incompatibility [2]. Hypersensitive reaction plays significant roles in plant pathogenic resistance, and a lot of the resistance genes, listed on a web site with a catalogue of wheat gene symbols (http://www.shigen.nig.ac.jp/wheat/komugi/genes/symbolClassList.jsp) have been reported in Triticeae species. In wheat and its related crops, interspecific crossing is extensively used for integration of agronomically important genes from genetic resources. If hybrid incompatibility arises as a by-product of adaptive evolution, the abundant resistance genes might lie hiding in genome as candidates for gene-flow barriers. Indeed, *Ne2* has risen considerably in frequency over, apparently due to tight linkage with loci that confer effective rust fungus resistance [2]. To accelerate introgression of the important genes from relative species into crops, the molecular nature of the causal genes for hybrid lethal and weakness such as *Ne2* and *Nec1* should be clarified in future studies.

## Materials and Methods

### Plant materials

For crossing with the tetraploid wheat (*Triticum turgidum* L.) cultivar Langdon (Ldn), 122 accessions of *Aegilops tauschii* Coss. were used as pollen parent, and are listed in Table S1. Their passport data including geographical coordinates appear in previous reports [18], [25]. The triploid F_1_ progeny were grown and selfed to produce wheat synthetics. All synthetics were independently generated through unreduced gamete formation in each of the triploid F_1_ hybrids [14]. The synthetics thus contained the A and B genomes from Ldn and the diverse D genomes originating from the *Ae. tauschii* male parents. In this study, the triploid hybrids and synthetic hexaploid lines showing normal growth features are represented as wild type (WT).

### Measurement of morphological traits and statistical analysis

Seeds of the synthetic lines were sown in November, 2006, and plants were grown individually in pots arranged randomly. The 16 traits studied are listed in Table S3. All morphological traits for the synthetic lines were measured on the three earliest tillers of each plant. Mean values were calculated using data from four plants in each line. The data were statistically analyzed using JMP software ver. 5.1.2 (SAS Institute, Cary, NC, USA). The correlations among the morphological traits were estimated based on Pearson correlation coefficient values, and principal component analyses were conducted using JMP software.

### Mapping of the causal gene for type III necrosis

For mapping population, F_2_ plants between WT (synthetic wheat line of Ldn and KU-2075) and type III necrosis (synthetic wheat line of Ldn and KU-2828) hexaploid lines were used. For SSR genotyping, 40 cycles of PCR were performed using 2x Quick Taq HS DyeMix (TOYOBO, Osaka, Japan) and the following conditions: 30 s at 94°C, 30 s for annealing, and 30 s at 68°C. The last step was incubation for 1 min at 68°C. Information on SSR markers and their annealing temperature was provided from the National BioResource Project (NBRP) KOMUGI web site (http://www.shigen.nig.ac.jp/wheat/komugi/strains/aboutNbrpMarker.jsp). The PCR products were separated by 2% agarose or 13% non-denaturing polyacrylamide gels, and visualized under UV light after staining with ethidium bromide. For polyacrylamide gel electrophoresis, the high efficiency genome scanning (HEGS) system (Nihon Eido, Tokyo, Japan) was used according to Hori et al. [49]. Genetic mapping was performed using MAPMAKER/EXP version 3.0b software [50]. For mapping of the *Lr34* locus, the following primer set was designed: 5′-TGCGGCGATTCTATACTACT-3′ and 5′-CCGACATCAAGAACCTCC-3′. The PCR-amplified products were digested by the 4-bp cutting restriction enzyme *Taq*I, and the digests were separated by a 13% non-denaturing polyacrylamide gel.

### Microarray analysis

Total RNA was extracted from the 1st (lowest) leaves of WT and type III necrosis synthetic lines, grown at 23°C for 3 weeks, using an RNeasy Plant Mini kit (Qiagen, Hilden, Germany). A KOMUGI 38k oligonucleotide DNA microarray (Agilent Technologies, Santa Clara, CA, USA) was used for the microarray analysis, and the microarrays were supplied through the National BioResource Project-Wheat (Japan, www.nbrp.jp). The platform of the Gene Expression Omnibus (GEO) is GPL9805 on the National Center of Biotechnology Information (NCBI), and more detailed information on the 38k microarray in Kawaura et al. [33]. RNA quality was checked using an Agilent 2100 BioAnalyzer, and Cy3-labeled cRNA was synthesized from total RNA samples by a Quick Amp Labeling kit (Agilent Technologies). Hybridization of the Cy3-labeled cRNA to the microarrays and washing were performed using a Gene Expression Hybridization kit and Gene Expression Wash Pack (Agilent Technologies). The scanned images were analyzed with Feature Extraction Software 9.5 (Agilent Technologies) using default parameters to obtain background subtracted and spatially detrended Processed Signal intensities. Two independent experiments were conducted. According to the MIAME (http://www.mged.org/Workgroups/MIAME/miame.html) compliant, all the microarray data have been deposited in the NCBI GEO (http://www.ncbi.nlm.nih.gov/geo/) as GSE19613 including supplementary files, GSM489170, GSM489171, GSM489172 and GSM489173.

Functions of the probes and genes were predicted by both BLAST and BLASTx searches (*E* value <1e^−10^) against the database of the DNA Data Bank of Japan (DDBJ). The NBRP KOMUGI web site (http://www.shigen.nig.ac.jp/wheat/komugi/array/index.jsp) was also referred to for the functional identification of the proteins encoded by the probes. Protein Knowledgebase (UniProtKB; http://www.uniprot.org/) was used for the functional categorization of identified genes.

### RT-PCR and quantitative RT-PCR

To study gene expression patterns, RT-PCR and quantitative RT-PCR analysis were performed on hexaploid synthetic lines grown at 23°C with a 16 h photoperiod, and total RNA samples were extracted from 2-, 3-, and 4-week-old seedling leaves of three WT and three type III necrosis synthetic lines. First-strand cDNA was synthesized from 1 µg of DNase I-treated RNA sample in a 20 µL reaction solution with oligo-dT primers using a ReverTra Ace-α- kit (TOYOBO). The gene-specific primer sets for the RT-PCR analyses are listed in Table S7. The PCR-amplified products were separated by electrophoresis through a 1.5% agarose gel and stained with ethidium bromide. The *Actin* gene was used as an internal control.

Quantitative RT-PCR was performed using a Thermal Cycler Dice (TaKaRa Bio, Ohtsu, Japan). The rate of amplification by SYBR Premix Ex Taq II (TaKaRa Bio) was monitored according to the manufacturer's protocol. Results were obtained as 2^−ΔCt^, where Ct is the difference in the number of PCR cycles required to reach the log phase of amplification for the examined gene and for *actin*, and were presented as values relative to the transcript levels in 2-week-old seedling leaves of the WT line (synthetic wheat line of Ldn and KU-2059).

### Transmission electron microscopic observation

The seedlings of the synthetic wheat lines were grown at 23°C for 3 weeks. The leaf blade was cut into 1 mm^2^ pieces, and incubated in freshly prepared 5 mM CeCl_3_ and 50 mM 3-(N-morpholino)propanesulfonic acid at pH 7.2 for 1 h. The incubated leaf samples were pre-fixed in 2.5% (v/v) glutaraldehyde and 2% (v/v) paraformaldehyde with 0.1 M cacodylate (CAB) buffer (pH 7.2). After washing three times with CAB buffer, the leaf samples were post-fixed with 1% (w/v) osmium tetroxide in CAB buffer for 1 h, and then the fixed leaf samples were dehydrated and embedded in Epon 812 resin (Nisshin EM, Tokyo, Japan). The sample blocks were cut with a diamond knife (Diatome, Bienne, Switzerland) on a Reichert-Nissei Ultracut microtome (Leica AG, Vienna, Austria) to obtain ultrathin sections (90 nm), which were collected on copper grids (200 mesh). Sections were examined and photographed using a Hitachi-7100 transmission electron microscope (Hitachi, Tokyo, Japan) at an accelerating voltage of 75 kV. Three independently produced sections derived from two plants were used for analysis of ROS generation and determination of the survival rate. More than 90 mesophyll cells in a section were counted for detection of dead cells. Mesophyll cells in which collapse of organelles, plasmolysis and breakage of cell membranes occurred were identified as dead cells. For elemental mapping of the electron-dense deposits, an energy-filtered LEO 912AB Omega transmission electron microscope (Carl Zeiss, Jena, Germany) was used. The procedure for the elemental mapping was according to Shinogi et al. [36].

### Histochemical detection of ROS production

The 1st and 2nd leaves of WT and type III necrosis lines grown at 23°C for 3 weeks were stained by NBT (Nitroblue tetrazolium; Nacalai tesque, Kyoto, Japan) for O_2_^−^ detection. The leaf samples were infiltrated in 0.5% (w/v) NBT solution for 8 h, and then treated with 100% ethanol for chlorophyll removal.

### Detection of autofluorescence

The 1st leaves of WT and type III necrosis lines grown for 2, 3 and 4 weeks were used for observation of yellow autofluorescence. The leaf samples were fixed and washed in boiling lactophenol/ethanol (1∶2, vol/vol). Then, the leaf samples were observed with a WIB filter under an Olympus BX51 fluorescence microscope (Olympus, Tokyo, Japan).

### Photosynthetic activity

A portable fluorometer JUNIOR-PAM (Heinz Walz GmbH, Effeltrich, Germany) was used for measurement of the maximum photochemical quantum yield of photosystem II (*Fv/Fm*). The 1st leaves of WT and type III necrosis lines grown for 2, 3 and 4 weeks were incubated under dark conditions for one hour. After the dark treatment, *Fv/Fm* was measured at ten independent locations of each leaf blade according to the operation manual for the fluorometer.

## Supporting Information

Table S1 Accession numbers, origins, sublineages, and triploid F_1_ hybrid phenotypes of *Ae. tauschii* accessions used in this study.
(0.08 MB PDF)

Table S2 Geographical distribution of the triploid F_1_ hybrid phenotypes in the *Ae. tauschii* populations.
(0.06 MB PDF)

Table S3 Comparison of morphological traits among the WT and hybrid necrosis lines of synthetic hexaploid wheat.
(0.06 MB PDF)

Table S4 Morphological traits - Eigenvectors for PC1 and PC2.
(0.04 MB PDF)

Table S5 List of the defense-related genes for which expression levels were altered in the type III necrosis line as inferred by microarray analysis.
(0.05 MB PDF)

Table S6 List of the ROS alleviation and photosynthesis related genes for which expression levels were altered in type III necrosis lines as inferred by microarray analysis.
(0.05 MB PDF)

Table S7 Primer sets for RT-PCR analysis in this study.
(0.06 MB PDF)
