# Supplementary material for: Hypersensitive Response-Like Reaction Is Associated with Hybrid Necrosis in Interspecific Crosses between Tetraploid Wheat and Aegilops tauschii Coss
Source: PLoS One. 2010 Jun 25;5(6):e11326. doi: 10.1371/journal.pone.0011326 (PMC2892878; doi:10.1371/journal.pone.0011326)
Supplement: Table S1 — Accession numbers, origins, sublineages, and triploid F1 hybrid phenotypes of Ae. tauschii accessions used in this study. (0.08 MB PDF) [file pone.0011326.s001.pdf]

Table S1 Accession numbers, origins, sublineages, and triploid F_1 hybrid phenotypes of *Ae. tauschii* accessions used in this study

Accession number	Country	Lower taxon	Sublineage*	F_1 phenotype
KU-2627	Afghanistan	var. *typica*	1-1	type II necrosis
KU-2012	Afghanistan	var. *typica*	1-2	type II necrosis
KU-2016	Afghanistan	var. *typica*	1-2	type II necrosis
KU-2022	Afghanistan	var. *typica*	1-2	WT
KU-2025	Afghanistan	var. *typica*	1-2	type II necrosis
KU-2027	Afghanistan	var. *typica*	1-2	WT
KU-2028	Afghanistan	var. *anathera*	1-2	not determined
KU-2032	Afghanistan	var. *typica*	1-2	not determined
KU-2042	Afghanistan	var. *typica*	1-2	type II necrosis
KU-2043	Afghanistan	var. *typica*	1-2	type II necrosis
KU-2056	Afghanistan	var. *typica*	1-2	not determined
KU-2058	Afghanistan	var. *typica*	1-2	not determined
KU-2059	Afghanistan	var. *anathera*	1-2	WT
KU-2621	Afghanistan	var. *typica*	1-2	type II necrosis
KU-2633	Afghanistan	var. *typica*	1-2	type II necrosis
KU-2636	Afghanistan	var. *anathera*	1-2	not determined
KU-2638	Afghanistan	var. *typica*	1-2	type II necrosis
KU-2639	Afghanistan	var. *typica*	1-2	type II necrosis
PI 476874	Afghanistan	var. *typica*	1-2	WT
CGN10734	Armenia	var. *typica*	1-2	type III necrosis
IG126280	Armenia	var. *typica*	1-5	WT
IG126293	Armenia	var. *typica*	1-5	not determined
IG48747	Armenia	var. *typica*	1-5	not determined
KU-2810	Armenia	var. *typica*	1-5	WT
KU-2816	Armenia	var. *typica*	1-5	WT
KU-2824	Armenia	var. *typica*	1-5	WT
KU-2811	Armenia	var. *typica*	2-1	WT
IG47196	Azerbaijan	var. *typica*	1-3	type III necrosis
IG 47182	Azerbaijan	var. *typica*	2-2	severe growth abortion
KU-2801	Azerbaijan	var. *typica*	2-2	not determined
KU-2806	Azerbaijan	var. *typica*	2-2	not determined
CGN10732	Azerbaijan	var. *typica*	2-3	severe growth abortion
IG47188	Azerbaijan	var. *typica*	2-3	severe growth abortion
IG47202	Azerbaijan	var. *typica*	2-3	hybrid chlorosis
PI508262	China	var. *typica*	1-3	not determined
AT47	China	var. *typica*	1-6	WT
AT55	China	var. *typica*	1-6	WT
AT60	China	var. *typica*	1-6	not determined
AT80	China	var. *typica*	1-6	WT
PI508264	China	var. *typica*	1-6	not determined
KU-20-1	Dagestan	var. *typica*	2-1	hybrid chlorosis
IG 120866	Dagestan	var. *typica*	2-2	severe growth abortion
KU-2828	Georgia	var. *typica*	1-2	type III necrosis
KU-2826	Georgia	var. *typica*	1-3	type III necrosis
KU-2834	Georgia	var. *typica*	1-3	not determined
AE454	Georgia	var. *typica*	HGL17	WT
AE929	Georgia	var. *typica*	HGL17	WT
KU-2829A	Georgia	var. *typica*	HGL17	WT
IG48042	India	var. *typica*	1-2	WT
KU-2154	Iran	var. *typica*	1-2	type II necrosis
KU-2068	Iran	var. *typica*	1-3	type II necrosis
KU-2087	Iran	var. *typica*	1-3	type II necrosis
KU-2122	Iran	var. *typica*	1-4	type II necrosis
KU-2144	Iran	var. *typica*	1-5	WT
KU-2145	Iran	var. *typica*	1-5	type III necrosis
KU-2152	Iran	var. *typica*	1-5	WT
KU-2157	Iran	var. *typica*	1-5	WT
KU-20-8	Iran	var. *typica*	2-1	WT
KU-2069	Iran	var. *typica*	2-1	hybrid chlorosis
KU-2078	Iran	ssp. *strangulata* Eig.	2-1	WT
KU-2083	Iran	var. *typica*	2-1	WT
KU-2111	Iran	var. *typica*	2-1	hybrid chlorosis
KU-2118	Iran	var. *typica*	2-1	WT

KU-2124	Iran	var. *typica*	2-1	WT
KU-2126	Iran	var. *typica*	2-1	WT
KU-2155	Iran	var. *typica*	2-1	not determined
KU-2156	Iran	var. *typica*	2-1	WT
KU-20-10	Iran	var. *meyeri*	2-2	not determined
KU-2088	Iran	ssp. *strangulata* Eig.	2-2	WT
KU-2096	Iran	var. *typica*	2-2	WT
KU-2100	Iran	var. *meyeri*	2-2	WT
KU-2110	Iran	var. *typica*	2-2	severe growth abortion
KU-2160	Iran	var. *meyeri*	2-2	WT
KU-2074	Iran	ssp. *strangulata* Eig.	2-3	WT
KU-2075	Iran	ssp. *strangulata* Eig.	2-3	WT
KU-2076	Iran	ssp. *strangulata* Eig.	2-3	WT
KU-2077	Iran	ssp. *strangulata* Eig.	2-3	not determined
KU-2079	Iran	ssp. *strangulata* Eig.	2-3	WT
KU-2080	Iran	ssp. *strangulata* Eig.	2-3	WT
KU-2090	Iran	ssp. *strangulata* Eig.	2-3	WT
KU-2091	Iran	ssp. *strangulata* Eig.	2-3	WT
KU-2092	Iran	ssp. *strangulata* Eig.	2-3	WT
KU-2093	Iran	ssp. *strangulata* Eig.	2-3	WT
KU-2097	Iran	var. *typica*	2-3	WT
KU-2098	Iran	var. *typica*	2-3	WT
KU-2101	Iran	var. *typica*	2-3	WT
KU-2102	Iran	var. *typica*	2-3	WT
KU-2103	Iran	var. *typica*	2-3	WT
KU-2104	Iran	var. *typica*	2-3	WT
KU-2105	Iran	var. *typica*	2-3	WT
KU-2106	Iran	var. *typica*	2-3	WT
KU-2108	Iran	var. *meyeri*	2-3	WT
KU-2158	Iran	var. *meyeri*	2-3	WT
KU-2159	Iran	var. *typica*	2-3	WT
AE1090	Kazakhstan	var. *typica*	1-4	WT
IG 131606	Kyrgyzstan	var. *typica*	1-4	WT
CGN10769	Pakistan	var. *typica*	1-1	type II necrosis
CGN10767	Pakistan	var. *typica*	1-2	type II necrosis
CGN10768	Pakistan	var. *typica*	1-2	WT
CGN10770	Pakistan	var. *typica*	1-2	WT
IG46663	Pakistan	var. *typica*	1-2	WT
IG46682	Pakistan	var. *typica*	1-4	not determined
KU-20-6	Pakistan	var. *anathera*	1-4	not determined
KU-2001	Pakistan	var. *typica*	1-4	type II necrosis
KU-2003	Pakistan	var. *anathera*	1-4	type II necrosis
IG47259	Syria	var. *typica*	1-4	WT
IG46623	Syria	var. *typica*	2-2	WT
IG48554	Tajikistan	var. *typica*	1-2	WT
AE1038	Tajikistan	var. *typica*	1-4	not determined
IG48559	Tajikistan	var. *typica*	1-4	not determined
KU-2136	Turkey	var. *typica*	1-2	WT
PI 554319	Turkey	var. *typica*	1-2	type II necrosis
KU-2132	Turkey	var. *typica*	1-5	WT
PI486277	Turkey	var. *typica*	1-5	not determined
PI 486267	Turkey	var. *typica*	2-1	not determined
IG48508	Turkmenistan	var. *typica*	1-1	not determined
IG126387	Turkmenistan	var. *typica*	1-2	WT
IG48518	Turkmenistan	var. *typica*	1-2	type II necrosis
IG126489	Turkmenistan	var. *typica*	1-5	type II necrosis
IG48539	Uzbekistan	var. *typica*	1-1	not determined
IG48567	Uzbekistan	var. *typica*	1-1	not determined
IG48565	Uzbekistan	var. *typica*	1-2	type II necrosis

*: Mizuno et al. [28]

KU: Plant Germ-Plasm Institute, Faculty of Agriculture, Kyoto University, Japan.

PI: National Small Grains Research Facility, USDA-ARS, USA.

IG: International Centre for Agricultural Research in the Dry Areas (ICARDA), Syria.

AE: Institut für Pflanzengenetik und Kulturpflanzenforschung (IPK), Germany.

AT: Faculty of Agriculture, Okayama University, Japan.
