# Supplementary material for: Hypersensitive Response-Like Reaction Is Associated with Hybrid Necrosis in Interspecific Crosses between Tetraploid Wheat and Aegilops tauschii Coss
Source: PLoS One. 2010 Jun 25;5(6):e11326. doi: 10.1371/journal.pone.0011326 (PMC2892878; doi:10.1371/journal.pone.0011326)
Supplement: Table S2 — Geographical distribution of the triploid F1 hybrid phenotypes in the Ae. tauschii populations. (0.06 MB PDF) [file pone.0011326.s002.pdf]

Table S2 Geographical distribution of the triploid F_1 hybrid phenotypes in the *Ae. tauschii* populations

	southwest Asia	central Asia	east China	middle-eastern Asia	Caspian	Caucasus	Total
WT	8	4	3	9	29	8	61
type III necrosis	0	0	0	1	0	4	5
type II necrosis	15	2	0	4	1	0	22
hybrid chlorosis	0	0	0	1	1	2	4
severe growth abortion	0	0	0	0	1	4	5
not determined	8	5	2	3	2	5	25
Total	31	11	5	18	34	23	122

Southwest and central Asia and east China are eastern habitats (≥60° longitude) of the *Ae. tauschii* accessions. Middle-eastern Asia, Caspian and Caucasus areas are western habitats (<60° longitude) of *Ae. tauschii*.
