# Supplementary material for: Hypersensitive Response-Like Reaction Is Associated with Hybrid Necrosis in Interspecific Crosses between Tetraploid Wheat and Aegilops tauschii Coss
Source: PLoS One. 2010 Jun 25;5(6):e11326. doi: 10.1371/journal.pone.0011326 (PMC2892878; doi:10.1371/journal.pone.0011326)
Supplement: Table S3 — Comparison of morphological traits among the WT and hybrid necrosis lines of synthetic hexaploid wheat. (0.06 MB PDF) [file pone.0011326.s003.pdf]

Table S3 Comparison of morphological traits among the WT and hybrid necrosis lines of synthetic hexaploid wheat

Morphological trait[a]	WT	type III necrosis
6th leaf blade length at the vegetative phase (cm)	25.84±4.91	18.07±9.45
6th leaf blade width at the vegetative phase (cm)*	0.74±0.11	0.53±0.13
Ratio of length to width in 6th leaf blade	34.72±4.45	39.19±2.96
Flag leaf length after heading (cm)	28.50±4.90	23.06±2.49
Flag leaf width after heading (cm)	1.03±0.11	0.81±0.27
Ratio of leaf to width in flag leaf	27.89±3.82	29.60±6.51
Length of leaf below the flag leaf (cm)	33.50±5.77	25.39±5.77
Width of leaf below the flag leaf (cm)*	0.91±0.09	0.75±0.20
Ratio of length to width in leaf below the flag leaf	36.83±4.63	37.73±4.69
Heading time (days)**	153.24±6.17	171.00±3.54
Flowering time (days)**	163.25±4.59	176.58±2.95
Culm length (cm)**	150.24±19.82	91.63±2.65
1st internode length (cm)**	57.17±7.48	25.24±3.22
2nd internode length (cm)**	28.46±4.11	14.03±2.52
3rd internode length (cm)**	21.50±3.64	11.18±0.90
Spike length (cm)	20.64±1.20	22.02±0.34
Seed number per spike**	25.41±6.23	12.95±0.06
Selfed seed fertility (%)**	84.36±15.68	39.17±8.25
Seed weight (1000 seeds, g)*	46.58±9.74	27.4±0.85

[a]Traits that show significant mean difference between WT and type III necrosis lines are indicated by * $P < 0.05$, or ** $P < 0.01$ (Student's *t* test).
