# Supplementary material for: Hypersensitive Response-Like Reaction Is Associated with Hybrid Necrosis in Interspecific Crosses between Tetraploid Wheat and Aegilops tauschii Coss
Source: PLoS One. 2010 Jun 25;5(6):e11326. doi: 10.1371/journal.pone.0011326 (PMC2892878; doi:10.1371/journal.pone.0011326)
Supplement: Table S4 — Morphological traits - Eigenvectors for PC1 and PC2. (0.04 MB PDF) [file pone.0011326.s004.pdf]

Table S4 Morphological traits - Eigenvectors for PC1 and PC2

Morphological trait	PC1	PC2
6th leaf blade length at the vegetative phase (cm)	0.24	0.20
6th leaf blade width at the vegetative phase (cm)	0.23	-0.04
Ratio of length to width of 6th leaf blade	0.01	0.39
Flag leaf length after heading (cm)	0.22	0.30
Flag leaf width after heading (cm)	0.28	-0.04
Ratio of leaf to width of flag leaf	0.00	0.44
Length of leaf below the flag leaf (cm)	0.28	0.23
Width of leaf below the flag leaf (cm)	0.29	0.01
Ratio of length to width of leaf below the flag leaf	0.15	0.34
Heading time (days)	-0.12	0.38
Flowering time (days)	-0.17	0.36
Culm length (cm)	0.31	-0.03
1st internode length (cm)	0.32	-0.03
2nd internode length (cm)	0.32	-0.05
3rd internode length (cm)	0.30	0.00
Spike length (cm)	0.01	0.05
Seed number per spike	0.21	-0.20
Selfed seed fertility (%)	0.19	-0.17
Seed weight (1000 seeds, g)	0.24	0.01
