# Supplementary material for: Hypersensitive Response-Like Reaction Is Associated with Hybrid Necrosis in Interspecific Crosses between Tetraploid Wheat and Aegilops tauschii Coss
Source: PLoS One. 2010 Jun 25;5(6):e11326. doi: 10.1371/journal.pone.0011326 (PMC2892878; doi:10.1371/journal.pone.0011326)
Supplement: Table S5 — List of the defense-related genes for which expression levels were altered in the type III necrosis line as inferred by microarray analysis. (0.05 MB PDF) [file pone.0011326.s005.pdf]

Table S5 List of the defense-related genes for which expression levels were altered in type III necrosis lines as inferred by microarray analysis

	up-regulated (>3-fold)		down-regulated (<1/3-fold)	
Gene	number of genes	mean Log2 ratio	number of genes	mean Log2 ratio
HR induced protein	1	7.7	0	-
dihydroflavonol 4-reductase	2	6.1	0	-
sesquiterpene cyclase	1	5.72	0	-
defensin	2	5.47	0	-
pathogenesis-related	9	5.46	0	-
flavanone-3-hydroxylase	4	5.28	0	-
germin	6	4.64	0	-
WCI-1	1	4.54	0	-
anthranilate N-benzoyltransferase	1	4.31	0	-
chalcone synthase	7	5.06	1	-2.22
wax protein	2	4	0	-
secretory protein	1	3.93	0	-
cinnamoyl-CoA reductase	5	3.85	0	-
flavonoid 7-O-methyltransferase	1	3.7	0	-
patatin	3	3.69	0	-
chalcone isomerase	2	3.46	0	-
esterase	3	3.13	0	-
phenylalanine ammonia-lyase	18	3.03	0	-
Hfr family	4	3.02	0	-
soluble inorganic pyrophosphatase	4	2.93	0	-
dioscorin class A	2	5.16	1	-1.6
chitinase	13	3.76	2	-3.79
harpin-induced 1	3	4.27	1	-2.04
MPI	5	2.68	0	-
thaumatin-like protein	5	2.67	0	-
WIR	4	2.5	0	-
caffeic acid O-methyltransferase	5	3.33	1	-2.89
cinnamyl alcohol dehydrogenase	3	2.26	0	-
lipoxygenase	15	2.12	0	-
Trans-cinnamate 4-monooxygenase	1	2.08	0	-
TPR-like domain containing protein	4	2.06	0	-
Multi antimicrobial extrusion protein	1	2.03	0	-
BRASSINOSTEROID INSENSITIVE 1-associated receptor kinase	2	1.95	0	-
naringenin,2-oxoglutarate 3-dioxygenase	1	1.85	0	-
proteinase inhibitor	8	3	2	-2.46

4-coumarate--CoA ligase	1	1.76	0	-
peroxidase	11	3.18	4	-2.46
cytochrome P450	14	3.12	5	-2.63
disease resistance gene	8	2.82	3	-1.71
quinone oxidoreductase	1	2.36	0	-
glutathione S-transferase	12	2.17	2	-2.99
xylanase inhibitor	3	3.01	1	-3.65
lipase	5	2.68	2	-2.09
L-ascorbate oxidase	2	4.68	2	-2.4
peptidase	6	2.5	4	-1.88
beta-1,3-glucanase	5	4.6	4	-4.23
serpin	1	5.44	2	-2.41
1-aminocyclopropane-1-carboxylate oxidase	1	2.11	1	-1.86
isopenicillin N synthase family protein	1	2.03	1	-3.19
O-methyltransferase	1	2.96	3	-2.52
collagenase	0	-	1	-1.69
extracellular invertase	0	-	1	-1.7
flower-specific gamma-thionin	0	-	1	-1.89
flavonol-3-O-glycoside-7-O-glucosyltransferase	0	-	1	-1.9
Total	221		46	
