# Supplementary material for: Hypersensitive Response-Like Reaction Is Associated with Hybrid Necrosis in Interspecific Crosses between Tetraploid Wheat and Aegilops tauschii Coss
Source: PLoS One. 2010 Jun 25;5(6):e11326. doi: 10.1371/journal.pone.0011326 (PMC2892878; doi:10.1371/journal.pone.0011326)
Supplement: Table S6 — List of the ROS alleviation and photosynthesis related genes for which expression levels were altered in type III necrosis lines as inferred by microarray analysis. (0.05 MB PDF) [file pone.0011326.s006.pdf]

Table S6 List of the ROS alleviation and photosynthesis related genes for which expression levels were altered in type III necrosis lines as inferred by microarray analysis

	up-regulated (>3-fold)		down-regulated (<1/3-fold)	
Gene	number of genes	mean log2 ratio	number of genes	mean log2 ratio
ROS alleviation related				
alternative oxidase	1	2.31	0	-
catalase	1	1.75	0	-
Photosynthesis related				
molecular mass early light-inducible protein	1	4.71	0	-
psbE	0	-	1	-1.66
ndhB	0	-	2	-1.72
chloroplast chlorophyll a/b binding protein	0	-	1	-1.8
psaB	0	-	1	-1.82
chloroplast cytochrome f	0	-	1	-1.88
psbB	0	-	1	-1.88
PSII 10-kD protein	0	-	2	-4.5
