# Supplementary material for: Hypersensitive Response-Like Reaction Is Associated with Hybrid Necrosis in Interspecific Crosses between Tetraploid Wheat and Aegilops tauschii Coss
Source: PLoS One. 2010 Jun 25;5(6):e11326. doi: 10.1371/journal.pone.0011326 (PMC2892878; doi:10.1371/journal.pone.0011326)
Supplement: Table S7 — Primer sets for RT-PCR analysis in this study. (0.06 MB PDF) [file pone.0011326.s007.pdf]

Table S7 Primer sets for RT-PCR analysis in this study

Gene	forward primer sequence (5'~3')	reverse primer sequence (5'~3')
hypersensitive-induced protein	ACTTAGGCTTAGGCAAATGGA	TAGCCATAAGCAAACATAGCC
chitinase a	CCATAGCCAACGCCGAG	GGCGTTATGGTTTTGGATGA
oxalate oxidase	TCATCAATTTTCCTGGCCTC	GAGCCTATTTGGGTTGGATG
6-STS	TGATGGGGTACGTCGGCGAG	TGGCGGAGCGGGATATGGA
2OG-Fe oxygenase	CCCAACGCCCTCACCATC	AGAGGAAGGAGGCGATGGAC
lipoxygenase	CATCCACACCATCACCAGCC	CAGCCCTTTCTCGCCGTC
CBL interacting protein kinase	CTCTCAACTGGACTCGACCT	ACATCACACCTTTCCTTCCA
UBQ-carboxyl terminal hydrolase	CTTTGCTCCAGTTGCTC	CATAATCATACGCACAGAAC
phosphate transporter	CAGGACCCGCACAAGCC	CAGGACCCGCACAAGCC
PR-1	GCAGAACTCGCCTCAGGACT	GCCGAGGTTATTGTTGCAGA
JA-induced protein	AGAAGGTGAGTGGGCTATTG	TGCCGCTGCTAACTGTG
PAL	GAAGACATTGAGCACCACCA	TTTGGTAGCACTGCACGG
WRKY3	GAGGGCGAGCACAACCACC	CTCCCGGCACACCTTCTTCA
peroxidase	TTTCACGATTGTTTTGTCAG	GATGACCTCAAAACCACG
R gene	CTCGACGACATGCCGGGAC	TTGACGCGCAGGCTGAGG
chloroplast ATP synthase CF1	TAGGAAATGATGCGGAACT	CCTCTTCCCTTTTTCTTTGT
PsbB	GTTTTGGTAGATGAGGAGGG	AGCAAAGCAAAGGTAGCAT
ethylene-responsive element binding protein	CCGACGACTCCGCCAACC	AAGGTGTGGGAGGGCGTCAA
Myb-like protein	CAGCAAATGGTCAATCC	ACAGCTTCACGCTTGTT
amine oxidase family protein	AAGATGATACCCGACGCCAC	CCCTTCCTCATCAGCAGCC
SAG	ACTCTGCTCCTCTGGCTTC	CCATTTTGCTTATTCAGTGC
PSII 10kD protein	GAAGGGTAGCCAAGGGGAAG	ATTTTGGTTTGCCGCACAG
Waox1a	TGCTCGACAAGATCGCCTACT	GGAGGCGCCAGTAGTCGAT
Waox1c	GGAGGCGTACACGTCGGACA	GTCATCAGGTGCATCCGCTC
PR-4	CGCGGTGAGCGCCTACTG	CCGATCCCGTTGGTGTCG
PR-5	TCGGCGGTGCTCTTCCTCCTC	GGCGACGTCGTTGGGGTGTT
Actin	GGCTGGTTTTGCTGGTGACGAAT	AATGAAGGAAGGCTGGAAGAGGA
